# Impact of maternal reproductive factors on cancer risks of offspring: A systematic review and meta-analysis of cohort studies

**DOI:** 10.1371/journal.pone.0230721

**Published:** 2020-03-30

**Authors:** Mi Ah Han, Dawid Storman, Husam Al-Rammahy, Shaowen Tang, Qiukui Hao, Gareth Leung, Maryam Kandi, Romina Moradi, Jessica J. Bartoszko, Callum Arnold, Nadia Rehman, Gordon Guyatt

**Affiliations:** 1 Department of Preventive Medicine, College of Medicine, Chosun University, Gwangju, Republic of Korea; 2 Department of Hygiene and Dietetics, Faculty of Medicine, Jagiellonian University Medical College, Krakow, Poland; 3 Life Sciences—Department of Biomedical and Molecular Sciences, Queen's University at Kingston, Kingston, Canada; 4 Department of Epidemiology, School of Public Health, Nanjing Medical University, Nanjing, Jiangsu, China; 5 The center of Gerontology and Geriatrics, National Center for Geriatric Clinical Research, West China Hospital, Sichuan University, Chengdu, Sichuan, China; 6 Department of Health Sciences, McMaster University, Hamilton, Ontario, Canada; 7 Department of Health Research Methods, Evidence and Impact, McMaster University, Hamilton, Ontario, Canada; 8 Division of Infectious Diseases, the Hospital for Sick Children, Toronto, Canada; 9 Department of Continuing Education, McMaster University, Hamilton, Canada; Norwegian Institute of Public Health, NORWAY

## Abstract

**Background:**

A number of studies have reported on associations between reproductive factors, such as delivery methods, number of birth and breastfeeding, and incidence of cancer in children, but systematic reviews addressing this issue to date have important limitations, and no reviews have addressed the impact of reproductive factors on cancer over the full life course of offspring.

**Methods:**

We performed a comprehensive search in MEDLINE, and Embase up to January 2020 and Web of Science up to 2018 July, including cohort studies reporting the association between maternal reproductive factors of age at birth, birth order, number of births, delivery methods, and breastfeeding duration and cancer in children. Teams of two reviewers independently extracted data and assessed risk of bias. We conducted random effects meta-analyses to estimate summary relative estimates, calculated absolute differences between those with and without risk factors, and used the GRADE approach to evaluate the certainty of evidence.

**Results:**

For most exposures and most cancers, we found no suggestion of a causal relation. We found low to very low certainty evidence of the following very small possible impact: higher maternal age at birth with adult multiple myeloma and lifetime uterine cervix cancer incidence; lower maternal age at birth with childhood overall cancer mortality (RR = 1.15, 95% CI = 1.01–1.30; AR/10,000 = 1, 95% CI = 0 to 2), adult leukemia and lifetime uterine cervix cancer incidence; higher birth order with adult melanoma, cervix uteri, corpus uteri, thyroid cancer incidence, lifetime lung, corpus uteri, prostate, testis, sarcoma, thyroid cancer incidence; larger number of birth with childhood brain (RR = 1.27, 95% CI = 1.06–1.52; AR/10,000 = 1, 95% CI = 0 to 2), leukemia (RR = 2.11, 95% CI = 1.62–2.75; AR/10,000 = 9, 95% CI = 5 to 14), lymphoma (RR = 4.66, 95% CI = 1.40–15.57; AR/10,000 = 11, 95% CI = 1 to 44) incidence, adult stomach, corpus uteri cancer incidence and lung cancer mortality, lifetime stomach, lung, uterine cervix, uterine corpus, multiple myeloma, testis cancer incidence; Caesarean delivery with childhood kidney cancer incidence (RR = 1.25, 95% CI = 1.01–1.55; AR/10,000 = 0, 95% CI = 0 to 1); and breastfeeding with adult colorectal cancer incidence.

**Conclusion:**

Very small impacts existed between a number of reproductive factors and cancer incidence and mortality in children and the certainty of evidence was low to very low primarily due to observational design.

## Introduction

Childhood cancer, one of the most frequent causes of death in children in developed countries, has increased over time [1]. The range of tumor types varies across age groups with leukemia most frequent in children aged 0 to 14 years and lymphoma in children aged 15 to 19 years [2].

Public health officials and investigators have recognized parental or family factors as possible determinants of childhood health. Many studies have reported that a variety of maternal reproductive factors, including delivery methods, age at birth, number of births, and breast feeding were associated with children’s adverse health outcomes [3–6]. Furthermore, scientists have raised concern regarding maternal risk factors that can influence the health status of offspring in adulthood [7–10].

The underlying mechanism of cancer risk in offspring according to the maternal reproductive factors involves the development of immune system. Cesarean delivery might interfere the development of the immune system through altering bacterial colonization or adverse birth stress response [11]. Breastmilk could influence the immune system with many immunologically active components [12]. Possible explanation for older mother relating cancer of offspring include transmission of altered DNA damage response and repair pathways in oocytes to the offspring [6].

Reproductive factors have, over the years, altered worldwide. Changes include increasing rate of cesarean delivery [13], earlier menarche [14, 15], and older age at birth [16]. These changes may have influenced health outcomes of children as well as mothers. Several systematic reviews have suggested an association between reproductive factors and cancer in children [12, 17, 18]. However, previous reviews have contained limitations including failure to assess the certainty of evidence [12, 18], to consider the magnitude of observed effects, or to address the full range of reproductive factors or relevant studies: some focused on specific types of reproductive factors [12, 19, 20], others on case-control studies [12, 17, 18], and few addressed the possible association between maternal reproductive factors and cancer risks in adulthood.

The purpose of this systematic review and meta-analysis was, therefore, to investigate the relation between maternal reproductive factors and cancer risks of children including the most recent studies reflecting women’s current reproductive behavior. Our perspective was to address the possibility that maternal reproductive factors are causally related to cancer incidence and mortality in children.

## Materials and methods

We conducted and reported this review in adherence to the Preferred Reporting Items for Systematic Review and Meta-analysis statement guidelines (S1 Table) [21]. We registered the protocol of this review at PROSPERO (CRD42018112045).

### Data source and search strategy

We developed a search strategy using a combination of controlled vocabulary (Mesh, Emtree terms) and free-text words related to reproductive factors and health outcomes (S2 Table). We performed a literature search in MEDLINE, and EMBASE from inception until January 2020 and Web of Science from inception until July 2018, consulted reference lists of included studies and systematic reviews for additional eligible studies, and made no restriction on publication period. This search is part of an effort to examine the relations between maternal reproductive factors and various health outcomes.

### Study selection

We included cohort studies with more than 1-year follow-up reporting the association between one or more reproductive factors and one or more cancer outcomes in children. Maternal reproductive factors included age at birth, birth order, total number of births, delivery methods and duration of breast-feeding. Cancer outcomes included total cancer incidence and mortality and major cancer types of childhood and adulthood: overall cancer, oral, esophagus, stomach, colorectum, liver, pancreas, larynx, lung, melanoma of skin, female breast, cervix uteri, corpus uteri, ovary, prostate, testis, kidney, urinary bladder, thyroid, brain and central nervous system, multiple myeloma, leukemia, lymphoma, eye, bone, and connective and soft tissue cancer.

Studies typically did not report associations with birth order, but if studies reported associations between number of older siblings or parity at birth and cancer, we inferred birth order from this information. Studies typically did not report associations with the total number of births, but if studies reported associations between the total number of children or siblings and cancer, we regarded them as the total number of births.

We excluded studies addressing associations between specific maternal health conditions (e.g., children born to mother with human immunodeficiency virus) and cancer, as well as reviews, case reports, or conference abstracts.

### Eligibility ascertainment and data extraction

Teams of two reviewers independently screened titles and abstracts of studies identified using the search strategy for potential eligibility, obtained full texts of any article that either reviewer believed might be eligible, and evaluated each full-text article for potential eligibility. Reviewers resolved disagreement by discussion or, if necessary, through adjudication by a third reviewer.

After calibration exercises to ensure validity and consistency, teams of two reviewers using pre-piloted extraction forms, independently extracted the following information from eligible studies: first author, publication year, country of origin, name of cohort; number of study participants, their age, gender, type and classification of reproductive factors; and cancer outcomes [type of cancer, age at assessment, follow-up period, effect estimates with corresponding 95% confidence interval (CI)]. Reviewers resolved disagreements by discussion or, if necessary, through adjudication by a third reviewer.

### Risk of bias

Teams of two reviewers independently assessed risk of bias using a modified version of the Quality in Prognostic Studies instrument with the following five items: (1) Study participation, (2) Study attrition, (3) Reproductive factor measurement, (4) Outcome measurement, and (5) Confounding [22]. Reviewers rated each item as low, moderate, or high risk and overall risk of bias as high if two or more items proved to be at a high risk of bias. Reviewers resolved disagreements through discussion or, if necessary, by adjudication form a third reviewer. S3 Table presents the instrument and the associated detailed guidance.

### Analysis

We performed meta-analyses, calculating summary relative effects of each reproductive factor on cancer incidence or mortality of children using the DerSimonian-Laird random-effects model. We visualized heterogeneity with forest plots and tested with I^2^ values and Q statistics. We performed statistical analyses using the R software 3.5.1 (the R foundation).

For maternal age at birth, we calculated pooled relative effects for highest and lowest age compared to 24 to 29 years. If a study did not explicitly consider an age category of 24 to 29, we chose the closest age category as the reference. We used age at birth between 24 to 29 years old as the reference group because it has been, over the past few decades, the most frequent age group of births in many countries [23] and many consider this as the optimal age of birth [24]. For birth order, total number of births, and duration of breastfeeding, we compared highest versus lowest categories. For delivery method, we calculated pooled relative effects comparing Cesarean with vaginal delivery.

For studies in which authors treated exposure as a continuous variable and did not present categorical analysis, we converted the relative effects estimate to correspond to a 10-year difference for maternal age at childbirth, 3 order difference for birth order, 3 degree difference for number of childbirths, in each case the most often reported difference, and used these in our meta-analyses.

We pooled risk estimates according to the life course of offspring based on when the outcomes were measured: childhood, adulthood, and lifetime. We classified the outcomes as childhood if a study assessed outcomes between 0 to 19 years, adulthood if a study assessed outcome 20 or more years and lifetime if a study assessed outcome in both childhood and adulthood. We could not calculate pooled estimates across lifetime due to the small number of eligible studies.

We conducted a priori specified meta-regression to test for differences between studies at higher versus lower risk of bias. We had planned to perform the Hartung-Knapp (HK) random meta-analysis as a sensitivity analysis and also planned to conduct subgroup analyses on income level of countries, and order of publication. However, we could not complete these additional analyses due to the small number of included studies.

### Certainty of evidence

We assessed certainty of evidence taking a causal perspective for each cancer risk using the Grading of Recommendations, Assessment, Development and Evaluation (GRADE) approach that uses four certainty categories (very low, low, moderate or high) [25]. One investigator assessed the certainty of evidence and a second investigator revised the certainty assessments as necessary. The GRADE approach to rating the certainty of evidence begins with the study design and then addresses five reasons to possibly rate down the certainty of evidence (risk of bias, imprecision, inconsistency of results, indirectness of evidence and publication bias) and three (including large magnitude of effect and dose-response gradient) to possibly rate up the certainty. When assessing causation, in the GRADE approach, randomized controlled trials (RCTs) start as high certainty evidence, while observational studies starts as low certainty evidence. If reasons for rating up are present (as in, for example, smoking as a causal exposure in lung cancer) certainty of evidence from observational studies can yield moderate or even high certainty evidence. We calculated absolute risk difference by multiplying relative effect and population risk of cancer, produced by the International Agency Research on Cancer (IARC) to estimate population cancer risks. IARC provided 5-year interval cumulative risk of cancer incidence or mortality using online analysis systems, we chose the median cumulative cancer risk among countries for 0 to 19 for childhood and for 20 to 74 for adulthood [26, 27]. For lifetime cancer risk, we used GLOBOCAN 2018 estimates of cumulative risk from 0 to 74 [28]. We presented summary of finding tables and stated plain language summary based on absolute effect magnitude and certainty of evidence [29].

## Results

### Study selection and characteristics of included studies

**Fig 1:**
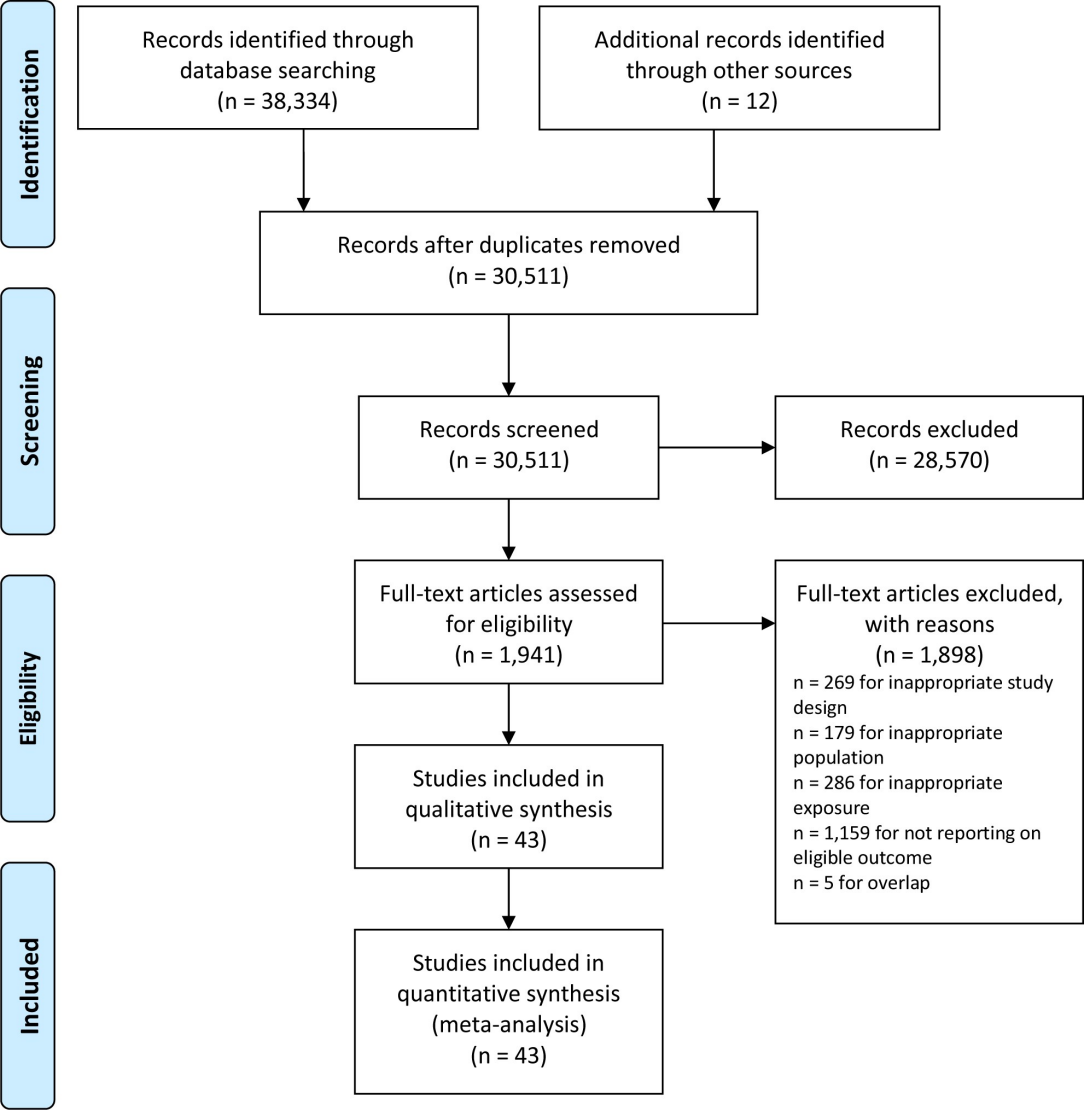
Search and selection of studies.

We identified 43 eligible studies including 24 cohorts enrolling more than 38 million participants (Fig 1, S4 Table) [7–10, 30–68]. Of these, 8 articles reported on overall cancer incidence, 2 on esophagus, 4 on stomach, 7 on colorectum, 4 on liver, 3 on pancreas, 1 on larynx, 4 on lung, 6 on melanoma, 11 on breast, 4 on cervix uteri, 5 on corpus uteri, 3 on ovary, 5 on prostate, 9 on testis, 8 on kidney, 2 on bladder, 3 on thyroid, 9 on brain, 3 on multiple myeloma, 14 on leukemia, 12 on lymphoma, 4 on eye, 4 on bone, 3 on connective and soft tissue cancer incidence. We also identified 3 articles reporting overall cancer mortality, 2 on stomach, 1 on colorectum, 1 on liver, 1 on lung, 2 on breast, 1 on prostate, 1 on brain, 1 on leukemia, 1 on lymphoma, 1 on eye, 1 on bone, and 1 on connective and soft tissue cancer mortality.

Of the eligible reports addressing cancer incidence, all addressing, liver, larynx, cervix uteri, kidney, bladder, thyroid, brain, multiple myeloma, leukemia, eye, bone, and connective and soft tissue cancer were at low risk of bias. This was also true of 1/2 on esophagus, 3/4 on stomach, 6/7 on colorectum, 2/3 on pancreas, 3/4 on lung, 5/6 on melanoma, 5/11 on breast, 4/5 on corpus uteri, 2/3 on ovary, 4/5 on prostate, 8/9 on testis cancer incidence and 11/12 on lymphoma. Of the eligible reports addressing cancer mortality, the following were all at low risk of bias: overall, colorectum, liver, prostate, brain, leukemia, lymphoma, eye, bone and soft tissue, as were 1/2 on stomach, 0/1 on lung, and 1/2 on breast cancer (S5 Table).

### Maternal reproductive factors and childhood cancer incidence and mortality

Table 1 presents the results for maternal reproductive factors in which we found an apparent impact [95% CI around relative risk (RR) excludes 1.0] on childhood cancer. These include lower maternal age at birth compared to 25 to 29 maternal age at birth and overall cancer mortality (RR = 1.15, 95% CI = 1.01–1.30; AR/10,000 = 1, 95% CI = 0 to 2; low certainty); a larger number of births and childhood brain cancer (RR = 1.27, 95% CI = 1.06–1.52; AR/10,000 = 1, 95% CI = 0 to 2; low certainty), leukemia (RR = 2.11, 95% CI = 1.62–2.75; AR/10,000 = 9, 95% CI = 5 to 14; low certainty) and lymphoma incidence (RR = 4.66, 95% CI = 1.40–15.57; AR/10,000 = 11, 95% CI = 1 to 44; very low certainty) compared to a smaller number of births. Compared to vaginal delivery, Caesarean delivery was associated with childhood kidney cancer incidence (RR = 1.25, 95% CI = 1.01–1.55; AR/10,000 = 0, 95% CI = 0 to 1; low certainty). The absolute magnitude of apparent impact in each case was very small: from 0 in 10,000 to 11 in 10,000. S6 Table presents the more frequent results: failure to establish an apparent impact of reproductive factors on cancer incidence or mortality (i.e. 95% CI around RR overlapping 1.0). S7 Table presents further details of results.

**Table 1:** Summary of finding for maternal reproductive factors and childhood cancer incidence and mortality.

Outcomes (no of studies)	No of cases/participants, follow-up years	Relative risk (95% CI)	Population risk (per 10,000) ^a^	Risk difference (per 10,000)	Certainty of the evidence	Plain language summary
**Lower maternal age at birth compared to 25 to 29 maternal age**						
Overall cancer mortality (1)	1114/NR, Mean 6.6	1.15 (1.01–1.30)	6	1 more (0 fewer to 2 more)	LOW (due to observational design)	Lower maternal age at birth may have little or no effect on overall cancer mortality
**Higher number of births compared to smaller number of births**						
Brain and CNS cancer incidence (1)	424/NR, Up to 14	1.27 (1.06–1.52)	4	1 more (0 fewer to 2 more)	LOW (due to observational design)	Higher number of births may have little or no effect on brain and CNS cancer incidence
Leukemia incidence (1)	306/NR, Up to 15	2.11 (1.62–2.75)	8	9 more (5 more to 14 more)	LOW (due to observational design)	Higher number of births may result in a very small increase in leukemia incidence
Lymphoma incidence (1)	13/NR, Mean 14.9	4.66 (1.40–15.57)	3	11 more (1 more to 44 more)	VERY LOW (due to observational design, imprecision) ^b^	We are uncertain of the effects of higher number of births on lymphoma incidence
**Cesarean delivery compared to vaginal delivery**						
Kidney cancer incidence (1)	717/6,907,253, Up to 14	1.25 (1.01–1.55)	1	0 fewer (0 fewer to 1 more)	LOW (due to observational design)	Cesarean delivery may have little or no effect on kidney cancer incidence

CI, Confidence Interval; CNS, Central Nervous System; NR, Not Reported

^a^ Cumulative risk between 0 to 19 years from the International Agency Research on Cancer online analysis system.

^b^ Confidence interval around absolute effect includes both no appreciable effect and appreciable harm.

### Maternal reproductive factors and adult cancer incidence and mortality

Table 2 presents the results for maternal reproductive factors in which we found an apparent impact (95% CI around RR excludes 1.0) on cancer during adulthood. These include higher maternal age at birth with adult multiple myeloma incidence (very low certainty); lower maternal age at birth compared to 25 to 29 age at birth with adult leukemia incidence (very low certainty); higher birth order with on adult melanoma (low certainty), cervix uteri (low certainty), corpus uteri (low certainty), and thyroid (low certainty) cancer incidence compared to lower birth order; larger number of births with adult stomach (low certainty), corpus uteri (low certainty) cancer incidence and lung cancer mortality (very low certainty). The absolute magnitude of apparent impact in each case was very small: from 3 in 1,000 to 21 in 1,000. S8 Table presents the more frequent results: failure to establish an apparent impact of reproductive factors on cancer incidence or mortality (i.e. 95% CI around RR overlapping 1.0). S9 Table presents additional detailed results.

**Table 2:** Summary of finding for maternal reproductive factors and adult cancer incidence and mortality.

Outcomes (no of studies)	No of cases/participants, follow-up years	Relative risk (95% CI)	Population risk (per 1,000) ^a^	Risk difference (per 1,000)	Certainty of the evidence	Plain language summary
**Higher maternal age at birth compared to 25 to 29 maternal age**						
Multiple myeloma incidence (1)	37/NR, Mean 11	4.53 (1.35–15.16)	4	14 more (1 more to 56 more)	VERY LOW (due to observational design, imprecision) ^b^	We are uncertain of the effects of higher maternal age at birth on multiple myeloma incidence
**Lower maternal age at birth compared to 25 to 29 maternal age**						
Leukemia incidence (1)	47/NR, Mean 11	2.08 (1.05–4.12)	6	6 more (0 fewer to 19 more)	VERY LOW (due to observational design, imprecision) ^b^	We are uncertain of the effects of higher maternal age at birth on leukemia incidence
**Higher birth order compared to lower birth order**						
Melanoma incidence (1)	7084/11314910, Up to 45	0.66 (0.53–0.80)	10	3 fewer (5 fewer to 2 fewer)	LOW (due to observational design)	Higher birth order may result in a very small decrease on melanoma incidence
Cervix uteri cancer incidence (1)	1292/11314910, Up to 45	0.57 (0.36–0.91)	7	3 fewer (4 fewer to 1 fewer)	LOW (due to observational design)	Higher birth order may result in a very small decrease on cervix uteri cancer incidence
Corpus uteri cancer incidence (1)	4490/11314910, Up to 45	0.66 (0.57–0.78)	16	5 fewer (7 fewer to 4 fewer)	LOW (due to observational design)	Higher birth order may result in a very small decrease on corpus uteri cancer incidence
Thyroid cancer incidence (1)	890/11314910, Up to 45	0.49 (0.26–0.91)	6	3 fewer (4 fewer to 1 fewer)	LOW (due to observational design)	Higher birth order may result in a very small decrease on thyroid cancer incidence
**Higher number of births compared to smaller number of births**						
Gastric cancer incidence (1)	1262/5657455, Up to 45	1.60 (1.06–2.41)	7	4 more (0 fewer to 10 more)	LOW (due to observational design)	Higher number of births may have little or no effect on gastric cancer incidence
Corpus uteri cancer incidence (1)	2966/5657455, Up to 45	0.47 (0.37–0.59)	16	8 fewer (10 fewer to 7 fewer)	LOW (due to observational design)	Higher number of births may result in a very small decrease in gastric cancer incidence
Lung cancer mortality (1)	67/1,272, Up to 25	2.18 (1.05–4.52)	18	21 more (1 more to 63 more)	VERY LOW (due to observational design, risk of bias, imprecision) ^b^	We are uncertain of the effects of higher number of births on lung cancer mortality
**Breastfeeding compared to no breastfeeding**						
Colorectal cancer incidence (1)	8651/548741, Mean 12.7	1.18 (1.12–1.24)	20	4 more (2 more to 5 more)	VERY LOW (due to observational design, risk of bias) ^c^	We are uncertain of the effects of breastfeeding on colorectal cancer incidence

CI, confidence interval; NR, not reported

^a^ Cumulative risk between 20 to 74 years the International Agency Research on Cancer online analysis system.

^b^ Confidence interval around absolute effect includes both no appreciable effect and appreciable harm.

^c^ Study at high risk of bias for inappropriate measurement of reproductive factor and inadequate adjustment for confounders

### Maternal reproductive factors and lifetime cancer incidence and mortality

Table 3 presents the results for maternal reproductive factors in which we found an apparent impact (95% CI around RR excludes 1.0) on lifetime cancer incidence. These include higher maternal age at birth with uterine cervix cancer incidence (low certainty); lower maternal age compared to 25 to 29 age at birth with uterine cervix cancer incidence (low certainty); higher birth order compared to lower birth order with increase in lung (low certainty), corpus uteri (low certainty), prostate (low certainty), testis (low certainty), connective and soft tissue (low certainty), thyroid (low certainty) cancer incidence; more compared to fewer number of births with stomach (low certainty), lung (low certainty), melanoma (low certainty), uterine cervix (low certainty), uterine corpus (low certainty), multiple myeloma (low certainty), and testis (low certainty) cancer incidence. The absolute magnitude of apparent impact in each case was very small: from 0 in 1,000 to 14 in 1,000. S10 Table presents the more frequent results: failure to establish an apparent impact of reproductive factors on cancer incidence or mortality (i.e. 95% CI around RR overlapping 1.0). S11 Table presents further detailed results.

**Table 3:** Summary of finding for maternal reproductive factors and lifetime cancer incidence and mortality.

Outcomes (no of studies)	No of cases/participants, follow-up years	Relative risk (95% CI)	Population risk (per 1,000) ^a^	Risk difference (per 1,000)	Certainty of the evidence	Plain language summary
**Higher maternal age at birth compared to 25 to 29 maternal age**						
Uterine cervix cancer incidence (2)	>25 />159,721, Up to 22	0.66 (0.51–0.85)	14	5 fewer (7 fewer to 2 fewer)	LOW (due to observational design)	Higher maternal age at birth may result in a very small decrease in uterine cervix cancer incidence
**Lower maternal age at birth compared to 25 to 29 maternal age**						
Uterine cervix cancer incidence (2)	>203/>223,133, Up to 22	1.61 (1.25–2.07)	14	9 more (4 more to 15 more)	LOW (due to observational design)	Lower maternal age at birth may result in a very small increase in cervix cancer incidence
**Higher birth order compared to lower birth order**						
Lung cancer incidence (1)	13174/11314910, Up to 45	1.26 (1.06–1.44)	28	7 more (2 more to 12 more)	LOW (due to observational design)	Higher birth order may result in a very small increase in lung cancer incidence
Corpus uteri cancer incidence (1)	NR/NR, Up to 46	0.60 (0.46–0.78)	10	4 fewer (5 fewer to 2 fewer)	LOW (due to observational design)	Higher birth order may result in a very small decrease in uterine corpus cancer incidence
Prostate cancer incidence (1)	NR/NR, Up to 46	1.38 (1.23–1.55)	37	14 more (9 more to 20 more)	LOW (due to observational design)	Higher birth order may result in a very small increase in prostate cancer incidence
Testis cancer incidence (2)	NR/>248,828, Up to 46	0.72 (0.58–0.89)	1	0 fewer (0 fewer to 0 fewer)	LOW (due to observational design)	Higher birth order may have little or no effect on testis cancer incidence
Thyroid cancer incidence (1)	4320/11,314,910, Up to 45	0.61 (0.47–0.78)	7	3 fewer (4 fewer to 2 fewer)	LOW (due to observational design)	Higher birth order may result in a very small decrease in thyroid cancer incidence
Connective and soft tissue cancer incidence (1)	3126/11,314,910, Up to 45	0.73 (0.55–0.97)	2	1 fewer (1 fewer to 0 fewer)	LOW (due to observational design)	Higher birth order may have little or no effect on connective and soft tissue cancer incidence
**Higher number of births compared to smaller number of births**						
Stomach cancer incidence (1)	946/NR, Up to 46	1.48 (1.31–1.67)	13	6 more (4 more to 9 more)	LOW (due to observational design)	Higher number of births may result in a very small increase in gastric cancer incidence
Lung cancer incidence (1)	3206/NR, Up to 46	1.13 (1.02–1.25)	28	4 more (1 more to 7 more)	LOW (due to observational design)	Higher number of births may result in a very small increase in lung cancer incidence
Melanoma incidence (2)	3630/NR, Up to 46	0.72 (0.65–0.79)	4	1 fewer (1 fewer to 1 fewer)	LOW (due to observational design)	Higher number of births may result in a very small decrease in melanoma incidence
Uterine cervix cancer incidence (1)	1450/NR, Up to 46	1.19 (1.08–1.31)	14	3 more (1 more to 4 more)	LOW (due to observational design)	Higher number of births may result in a very small increase in uterine cervix cancer incidence
Uterine corpus cancer incidence (1)	1858/NR, Up to 46	0.76 (0.70–0.82)	10	2 fewer (3 fewer to 2 fewer)	LOW (due to observational design)	Higher number of births may result in a very small decrease in uterine corpus cancer incidence
Testis cancer incidence (3)	>1,030/>222,769.84, Up to 46	0.73 (0.64–0.83)	1	0 fewer (0 fewer to 0 fewer)	LOW (due to observational design)	Higher number of births may have little or no effect on testis cancer incidence
Multiple myeloma incidence (1)	680/NR, Up to 45	1.34 (1.08–1.66)	2	1 more (0 fewer to 1 more)	LOW (due to observational design)	Higher number of births may have little or no effect on multiple myeloma incidence

CI, confidence interval; NR, not reported

^a^ Lifetime cumulative risk from Globocan 2018 statistics (Farley et al., 2019).

## Discussion

We found evidence of a number of associations between maternal reproductive factors and cancer incidence and mortality in children: higher maternal age at birth with adult multiple myeloma and lifetime uterine cervix cancer incidence; lower maternal age at birth with childhood overall cancer mortality, adult leukemia and lifetime uterine cervix cancer incidence; higher birth order with adult melanoma, cervix uteri, corpus uteri, thyroid cancer incidence, lifetime lung, corpus uteri, prostate, testis, connective and soft tissue, thyroid cancer incidence; larger number of births with childhood brain, leukemia, lymphoma incidence, adult stomach, corpus uteri cancer incidence and lung cancer mortality, lifetime stomach, lung, uterine cervix, uterine corpus, multiple myeloma, testis cancer incidence; and Caesarean delivery with childhood kidney cancer incidence. As presented in Tables 1 to 3, the magnitude of the apparent effects in absolute terms was, however, very small. Further, the certainty of the evidence in terms of establishing a causal relation between exposure and outcomes was low or very low.

The strengths of this review included our decision to focus on causation, and our use of GRADE assessment for the certainty of evidence. Moreover, we calculated the magnitude of the effects not only with relative, but also with absolute effects. Teams of two reviewers judged eligibility and independently extracted data and assessed risk of bias utilizing, if needed, third party adjudication. We conducted the analyses according to the developmental period of offspring, presenting results from both childhood and from adulthood. This provided a comprehensive picture of the associations between maternal reproductive factors on cancer risks of children throughout their lives.

Turning to limitations, our review was based on observational studies that provided only low or very low certainty evidence for causation. Although RCTs could provide a high certainty of evidence for causality, the nature of the exposures of interest makes RCTs unfeasible or unethical or practical reasons, except for highly specific situations such as Caesarean section delivery for breech presentation [69] or promotion programs for breast feeding [70].

We had planned to perform the Hartung-Knapp (HK) random meta-analysis as a sensitivity analysis but could not present due to a small number of studies included [71, 72]. Also, we could not perform prior planned subgroup analysis such as income level of countries or order of publication due to the small number of eligible studies.

Some previous studies reported differential effects of cancer subtype according to maternal reproductive factors such as maternal age effects for childhood lymphoblastic vs myeloid leukemia [33], or sibship size and Hodgkin vs non-Hodgkin lymphoma [57]. Our failure to plan and conduct analyses for the cancer subtype therefore represents a limitation of our work.

Most eligible studies used existing databases, such as birth registries that provided representative and nearly complete data for the target population with limited risk for selection and attrition bias. Sometimes registers contained only limited confounder information and primary studies could, as a result, not address some important confounders [73]. Because registry-based studies often have great statistical power to detect small effects, they may highlight unimportant effects. We dealt with this issue by calculating and presenting absolute as well as relative risks. In doing so, we found that the magnitude of any possible impact of reproductive factors on cancer was extremely small.

Our results are consistent with previous reviews addressing the association between maternal reproductive factors and offspring’s cancer risk with regard to relative risks. However, the majority of previous reviews focused on case-control studies [12, 18] that are at a higher risk of bias than cohort studies [74, 75]. For case-control studies, differential ascertainment of exposure and outcomes in cases and controls could cause bias. Nested-case control could measure variables in the same way as cohort studies and, when performed appropriately, thus prevent differential assessment. They remain vulnerable, however, to differential participation due to their procedure for selecting cases and controls from the defined cohort. Although the studies included in this review also relied on exposure information reported by mothers or fathers, differential recall bias is likely to be far less in cohort studies that address exposure before outcomes occur [45, 64]. Cohort studies also provide the most representative results of the population of interest.

Prior investigators have not, as our review has done (Tables 2 and 3 and S8 and S10 Tables) summarized the effect of maternal reproductive factors on cancer risk according to the life course of offspring. Moreover, prior reviews did not address possible absolute impact of reproductive factors on cancer incidence and mortality.

Had we addressed association alone, our certainty of evidence would have been in most cases high to moderate. We chose, however, to address possible causal relationships, and the possibility of residual confounding in observational studies limits inferences regarding causation. For instance, even when associations exist, they may be due not to the maternal reproductive factors, but other factors such as socioeconomic status and maternal education that are themselves associated with maternal reproductive factors. Were this the case, modification of reproductive factors, even when associated with cancer outcomes, would have no impact on those outcomes. Our GRADE ratings of low to very low certainty evidence reflect our goal of addressing causation.

## Conclusion

Thus, low to very low certainty of evidence suggests a causal link between a number of maternal reproductive factors and several types of cancer in offspring. Even were decision makers prepared to suggest public health initiatives to modify cancer risk on the basis of low to very low certainty evidence of causation, however, our results would provide only a very limited rationale for such action. The reason is that the magnitude of any absolute effects of the exposure was small to very small. Public health decision makers interested in reducing cancer in children–either during childhood or adulthood–should, therefore, focus on areas other than maternal reproductive factors.

## Supporting information

S1 Table PRISMA checklist. (DOC)

S2 Table Search strategy. (DOCX)

S3 Table Detailed guideline for assessment of risk of bias. (DOCX)

S4 Table Study characteristics of included studies. (DOCX)

S5 Table Risk of bias assessment of included studies. (DOCX)

S6 Table Summary of finding for maternal reproductive factors and childhood cancer incidence and mortality. (DOCX)

S7 Table Maternal reproductive factors and cancer incidence and mortality in childhood. (DOCX)

S8 Table Summary of finding for maternal reproductive factors and adult cancer incidence and mortality. (DOCX)

S9 Table Maternal reproductive factors and cancer incidence and mortality in adulthood. (DOCX)

S10 Table Summary of finding for maternal reproductive factors and lifetime cancer incidence and mortality. (DOCX)

S11 Table Maternal reproductive factors and cancer incidence and mortality in lifetime. (DOCX)
