# Supplementary material for: Impact of maternal reproductive factors on cancer risks of offspring: A systematic review and meta-analysis of cohort studies
Source: PLoS One. 2020 Mar 30;15(3):e0230721. doi: 10.1371/journal.pone.0230721 (PMC7105118; doi:10.1371/journal.pone.0230721)
Supplement: S2 Table — (DOCX) [file pone.0230721.s002.docx]

**S2 Table. Search Strategy**

Ovid MEDLINE(R) Epub Ahead of Print, In-Process & Other Non-Indexed Citations, Ovid MEDLINE(R) Daily and Ovid MEDLINE(R) 1946 to Present

Search Strategy:

| # | Searches |
| --- | --- |
| 1 | (reproductive adj (factor* or characteristic* or histor*)).tw,kf. |
| 2 | Cesarean Section/ or Labor, Induced/ or ((c?esarean adj (section* or deliver* or birth*)) or (Induction adj (deliver* or birth* or labor)) or (induced adj (labor or birth* or deliver*))).tw,kf. |
| 3 | Milk, Human/ or Breast Feeding/ or (breast adj (feed* or milk)).tw,kf. |
| 4 | Maternal Age/ or (age* adj (birth* or childbirth* or deliver* or parity or childbearing* or maternal or pregnanc* or mother*)).tw,kf. |
| 5 | Parity/ or Delivery, Obstetric/ or Pregnancy, Multiple/ or ((parity or pregnan* or child* or birth*) adj (histor* or number* or frequenc* or order*) or nullipar* or multiparous or multiparit*).tw,kf. |
| 6 | Menarche/ or (Menarche* adj age).tw,kf. |
| 7 | Or/1-6 |
| 8 | exp Risk/ or risk*.tw,kf. |
| 9 | Mortality/ or (death* or mortality or mortalities or fatality or fatalities).tw,kf. |
| 10 | exp Cardiovascular Diseases/ or (cardiovascular adj (disease* or mortalit* or incidence*)).tw,kf. |
| 11 | Stroke/ or Cerebral Hemorrhage/ or (stroke* or (cerebral adj h?emorrhage)).tw,kf. |
| 12 | Coronary Artery Disease/ or Coronary Disease/ or Myocardial Infarction/ or Myocardial Ischemia/ or ((coronary adj disease*) or (isch?emic adj heart) or (myocardial adj (infarct* or isch?emi*)) or angina*).tw,kf. |
| 13 | Diabetes Mellitus/ or Diabetes Mellitus, Type 1/ or Diabetes Mellitus, Type 2/ or diabet*.tw,kf. |
| 14 | Obesity/ or Pediatric Obesity/ or obes*.tw,kf. |
| 15 | Hypertension/ or (hypertension or hypertens*).tw,kf. |
| 16 | exp Neoplasms/ or (cancer* or neoplas* or tumo?r* or malignan*).tw,kf. |
| 17 | exp Inflammatory Bowel Diseases/ or ((ulcer* adj coli*) or crohn or (inflam* adj bowel* adj (disease* or disorder*))).tw,kf. |
| 18 | Asthma/ or asthma.tw,kf. |
| 19 | Dermatitis, Atopic/ or (atop*).tw,kf. |
| 20 | Arthritis, Rheumatoid/ or (rheumatoid adj arthriti*).tw,kf. |
| 21 | exp Autism Spectrum Disorder/ or (autis* or asperger*).tw,kf. |
| 22 | Attention Deficit Disorder with Hyperactivity/ or (ADHD or (attention adj deficit*) or hyperactivit* or inatten*).tw,kf. |
| 23 | or/9-22 |
| 24 | exp cohort studies/ or ((cohort adj (study or studies)) or cohort analy*).tw,kf. |
| 25 | (follow up adj (study or studies)).tw,kf. |
| 26 | (longitudinal or prospective or retrospective).tw,kf. |
| 27 | or/24-26 |
| 28 | 7 and 8 and 23 and 27 |

Embase Classic+Embase 1974 to 2020 Jan 6

Search Strategy:

| # | Searches |
| --- | --- |
| 1 | (reproductive adj (factor* or characteristic* or histor*)).tw,kw. |
| 2 | Cesarean Section/ or Labor Induction/ or ((c?esarean adj (section* or deliver* or birth*)) or (Induction adj (deliver* or birth* or labor)) or (induced adj (labor or birth* or deliver*))).tw,kw. |
| 3 | Breast Milk/ or Breast Feeding/ or (breast adj (feed* or milk)).tw,kw. |
| 4 | Maternal Age/ or (age* adj (birth* or childbirth* or deliver* or parity or childbearing* or maternal or pregnanc* or mother*)).tw,kw. |
| 5 | Parity/ or Obstetric Delivery/ or Pregnancy, Multiple/ or ((parity or pregnan* or child* or birth* or childbearing) adj (histor* or number* or frequenc* or order*) or nullipar* or multiparous or multiparit*).tw,kw. |
| 6 | Menarche/ or (Menarche* adj age).tw,kw. |
| 7 | Or/1-6 |
| 8 | exp Risk/ or risk*.tw,kw. |
| 9 | Mortality/ or (death* or mortality or mortalities or fatality or fatalities).tw,kw. |
| 10 | exp Cardiovascular Diseases/ or (cardiovascular adj (disease* or mortalit* or incidence*)).tw,kw. |
| 11 | Stroke/ or Cerebral Hemorrhage/ or (stroke* or (cerebral adj h?emorrhage)).tw,kw. |
| 12 | Coronary Artery Disease/ or Coronary Disease/ or Myocardial Infarction/ or Myocardial Ischemia/ or ((coronary adj disease*) or (isch?emic adj heart) or (myocardial adj (infarct* or isch?emi*)) or angina*).tw,kw. |
| 13 | Diabetes Mellitus/ or Diabetes Mellitus, Type 1/ or Diabetes Mellitus, Type 2/ or diabet*.tw,kw. |
| 14 | Obesity/ or Pediatric Obesity/ or obes*.tw,kw. |
| 15 | Hypertension/ or (hypertension or hypertens*).tw,kw. |
| 16 | exp Neoplasms/ or (cancer* or neoplas* or tumo?r* or malignan*).tw,kw. |
| 17 | exp Inflammatory Bowel Diseases/ or ((ulcer* adj coli*) or crohn or (inflam* adj bowel* adj (disease* or disorder*))).tw,kw. |
| 18 | Asthma/ or asthma.tw,kw. |
| 19 | Dermatitis, Atopic/ or (atop*).tw,kw. |
| 20 | Arthritis, Rheumatoid/ or (rheumatoid adj arthriti*).tw,kw. |
| 21 | exp Autism Spectrum Disorder/ or (autis* or asperger*).tw,kw. |
| 22 | Attention Deficit Disorder with Hyperactivity/ or (ADHD or (attention adj deficit*) or hyperactivit* or inatten*).tw,kw. |
| 23 | or/9-22 |
| 24 | Longitudinal Study/ or Retrospective Study/ or Prospective Study/ or Cohort Analysis/ |
| 25 | (cohort adj (study or studies)).tw,kw. |
| 26 | (follow up adj (study or studies)).tw,kw. |
| 27 | (longitudinal or prospective or retrospective).tw,kw. |
| 28 | or/24-27 |
| 29 | 7 and 8 and 23 and 28 |

[Web of Science]

Science Citation Index Expanded (SCI-EXPANDED) --1900-present

Social Sciences Citation Index (SSCI) --1956-present

| Set | Searches |
| --- | --- |
| # 30 | #29 AND #23 AND #8 AND #7 |
| # 29 | #28 OR #27 OR #26 OR #25 OR #24 |
| # 28 | TS=(cohort NEAR (study or studies)) |
| # 27 | TS=("follow up" NEAR (study or studies)) |
| # 26 | TS=(longitudinal NEAR (study or studies)) |
| # 25 | TS=(observational NEAR(study or studies)) |
| # 24 | TS=(prospective NEAR (study or studies)) |
| # 23 | #22 OR #21 OR #20 OR #19 OR #18 OR #17 OR #16 OR #15 OR #14 OR #13 OR #12 OR #11 OR #10 OR #9 |
| # 22 | TS=(ADHD or (attention NEAR deficit*) or hyperactivit* or inatten*) |
| # 21 | TS=(autis* or asperger*) |
| # 20 | TS=(rheumatoid NEAR arthriti*) |
| # 19 | TS=(atop*) |
| # 18 | TS=asthma |
| # 17 | TS=((ulcer* NEAR coli*) or crohn or (inflam* NEAR bowel* NEAR (disease* or disorder*))) |
| # 16 | TS=(cancer* or neoplas* or tumo*r* or malignan*) |
| # 15 | TS=(hypertension or hypertens*) |
| # 14 | TS=obes* |
| # 13 | TS=diabet* |
| # 12 | TS=((coronary NEAR disease*) or (isch*emic NEAR heart) or (myocardial NEAR (infarct* or isch*emi*)) or angina*) |
| # 11 | TS=(stroke* or (cerebral adj h?emorrhage)) |
| # 10 | TS=(cardiovascular NEAR (disease* or mortalit* or incidence*)) |
| # 9 | TS=(death* or mortality or mortalities or fatality or fatalities) |
| # 8 | TS=(risk*) |
| # 7 | #6 OR #5 OR #4 OR #3 OR #2 |
| # 6 | TS=(Menarche* adj age) |
| # 5 | TS=((parity or pregnan* or child* or birth*) NEAR (histor* or number* or frequenc* or order*) or nullipar* or multiparous or multiparit*) |
| # 4 | TS=(age* NEAR (birth* or childbirth* or deliver* or parity or childbearing* or maternal or pregnanc* or mother*)) |
| # 3 | TS=(breast NEAR (feed* or milk)) |
| # 2 | TS=(((c*esarean NEAR (section* or deliver* or birth*)) or (Induction NEAR (deliver* or birth* or labor)) or (induced NEAR (labor or birth* or deliver*)))) |
| # 1 | TS=(reproductive NEAR (factor* or characteristic* or histor*)) |
