# Supplementary material for: Impact of maternal reproductive factors on cancer risks of offspring: A systematic review and meta-analysis of cohort studies
Source: PLoS One. 2020 Mar 30;15(3):e0230721. doi: 10.1371/journal.pone.0230721 (PMC7105118; doi:10.1371/journal.pone.0230721)
Supplement: S3 Table — (DOCX) [file pone.0230721.s003.docx]

**S3 Table. Detailed guideline for assessment of risk of bias**

| 1. Study participation | Goal: To judge the risk of selection bias (**likelihood that relationship between reproductive factors and outcome is different for participants and eligible non-participants**).   \| Low risk \| Exposed and unexposed groups are selected from the same population and enrolment in the study was independent of reproductive factor. For example, the study population includes all babies surviving their first year of living in Norway and Sweden 1984–2004.  Participants and non-participants are comparable; use randomized sampling methods or proper sampling frame with no significant difference in baseline characteristics between participants and non-participants. (Inclusion/exclusion criteria clearly reported, description of selection process such as recruitment period, place of recruitment, baseline characteristics). \| \| --- \| --- \| \| Moderate risk \| Recruitment process is vague or unclear recruitment \| \| High risk \| Systematic differences between participants and non-participants; if a study recruited study population, and then only analyzed people without missing data of exposure variables. If there is a meaningful difference of background characteristics between included and non-included people, this is suspicious situation of selection bias.  Participants and non-participants are not comparable. For example, a web-based birth cohort recruit people using internet, and study participants are limited to internet users.  Participant selection process are not reported. \| |
| --- | --- | --- | --- | --- | --- | --- | --- |
| 2. Study attrition (loss to follow-up) | Goal: To judge the risk of attrition bias (**likelihood that relationship between reproductive factor and outcome are different for completing and non-completing participants**).   \| Low risk \| At least 90% retention for the duration of the study. \| \| --- \| --- \| \| Moderate risk \| 80 to 89% retention for the duration of the study.  If rate of follow-up is not reported but the study is likely to have low rates of loss to follow-up through government or local databases and registries. For example, outcome could be ascertained from the National Death Index even if participants weren't actively responding to follow-up questionnaires. \| \| High risk \| Less than 80% follow-up.  Loss to follow-up is not reported or cannot be estimated from information provided in the paper. \| |
| 3. Reproductive factor measurement | Goal: To judge the risk of measurement bias related to how reproductive factor was measured (**differential measurement of reproductive factor related to the level of outcome**).   \| Low risk \| RF is measured by self-report of mother or primary caregiver less than 20 years duration of recall with or without external validation. Register-based study (e.g., birth statistics registries, medical birth registers) \| \| --- \| --- \| \| Moderate risk \| RF is measured by self-report of mother or primary caregiver more than 20 years duration of recall with or without external validation.  Medical record review without validation \| \| High risk \| RF is measured by self-report of offspring (children) or someone other than mother or primary caregiver. For example, participants are asked whether they were breastfed when they were infants.  No description of RF measurement. \| |
| 4. Outcome measurement | Goal: To judge the risk of bias related to the measurement of outcome (**differential measurement of outcome related to the baseline level of reproductive factor**).   \| Low risk \| Based on national or local registries with or without review by study physician or study staff (e.g., National Death Index, National Program of Cancer Registries (NPCR))  Medical records reviewed by a study physician or study staff  Multiple measures by investigators regardless of the criteria used to define this (e.g., blood pressure for hypertension, height/weight for obesity) \| \| --- \| --- \| \| Moderate risk \| Self-report with external validation by medical records. For example, a study may ask participants whether they have been diagnosed with breast cancer every two years. Medical records may be retrieved for participants who indicate a diagnosis of breast cancer. The authors may report that medical records confirmed self-report breast cancer for over 98% of cases.  External validation may only be done for a subsample of participants. This is acceptable as long as the rate of concordance between self-report and medical records is high (>95%).  Self-report with no external validation for all-cause mortality.  Single (one time point) measure by investigator regardless of the criteria used to define this (e.g., blood pressure for hypertension, height/weight for obesity) \| \| High risk \| Medical records without review by study physician or study staff.  Self-report with no external validation.  The authors do not specify how outcomes were measured. \| |
| 5. Study confounding | Goal: To judge the risk of bias due to confounding (**i.e. the effect of reproductive factor is distorted by another factor that is related to reproductive factor and outcome**).   \| **Adult offspring** \| \| \| --- \| --- \| \| Low risk \| The study adjusts at a minimum for age, sex (only for adult offspring), smoking, at least one measure of socioeconomic status such as level of income or education or occupation, family history (of cancer for cancer outcomes, cardiovascular disease for cardiovascular outcomes, diabetes for diabetes outcomes, hypertension for hypertension outcomes, psychiatric disease for mental health outcomes, autoimmune disease for immune associated outcomes, allergic/atopic disease for asthma/atopic dermatitis), aspirin use (necessary only for colon cancer), diabetes (necessary only for cardiovascular outcomes, excluding diabetes), alcohol consumption (only for cancers of the mouth, pharynx, larynx, esophagus, colorectum, and breast), weight or BMI (only for cancers of the esophagus, pancreas, liver, breast, endometrium) and physical activity (only for cardiovascular outcomes), at least one measure of weight for obesity outcome (birthweight of children, maternal pre-pregnancy bmi/weight /obesity, gestational weight gain) in the analysis. \| \| Moderate risk \| Adjusts at a minimum for age, sex (only for adult offspring), smoking, family history (of cancer for cancer outcomes, cardiovascular disease for cardiovascular outcomes, diabetes for diabetes outcomes, hypertension for hypertension outcomes, psychiatric disease for mental health outcomes, autoimmune disease for immune associated outcomes), and diabetes (only for cardiovascular outcomes, excluding diabetes). \| \| High risk \| Adjusts no more than age, and smoking. \|      \| **Child (offspring)** \| \| \| --- \| --- \| \| Low risk \| The study adjusts at a minimum for age (or gestational age), sex, at least one measure of parental (maternal or paternal) socioeconomic status such as level of income or education or occupation, family history (of cancer for cancer outcomes, diabetes for diabetes outcomes, psychiatric disease for mental health outcomes, autoimmune disease for immune associated outcomes, allergic/atopic disease for asthma/atopic dermatitis), smoking of mother, at least one measure of weight for obesity outcome (birthweight of children, maternal pre-pregnancy bmi/weight /obesity, gestational weight gain) in the analysis. \| \| Moderate risk \| Adjusts at a minimum for age (or gestational age), sex, family history (of cancer for cancer outcomes, diabetes for diabetes outcomes, psychiatric disease for mental health outcomes, autoimmune disease for immune associated outcomes) \| \| High risk \| Adjusts no more than age (or gestational age) and sex. \| |
