# Supplementary material for: Impact of maternal reproductive factors on cancer risks of offspring: A systematic review and meta-analysis of cohort studies
Source: PLoS One. 2020 Mar 30;15(3):e0230721. doi: 10.1371/journal.pone.0230721 (PMC7105118; doi:10.1371/journal.pone.0230721)
Supplement: S4 Table — (DOCX) [file pone.0230721.s004.docx]

**S4 Table. Study characteristics of included studies**

| **Author** | **Year** | **Cohort (s)** | **Country** | **Participants at baseline (n)** | **Age at baseline** | **% female** | **Duration of follow-up (years)** | **Type of reproductive factors** |
| --- | --- | --- | --- | --- | --- | --- | --- | --- |
| Barber et al.^1^ | 2019 | Black Women’s Health Study (BWHS) | United States | 59,000 | Mean: 48.31 | 100 | Up to: 11 | Birth order, maternal age at birth, breastfeeding duration |
| Martin, et al.^2^ | 2005 | Boyd Orr Cohort | United Kingdom | 4,999 | range: 0 to 19 | 51.01 | mean: 48.25 | breastfeeding duration |
| Lu, et al.^3^ | 2010 | California Teachers Study | United States | 110,999 | mean: 52.5 | 100 | mean: 11 | maternal age at birth |
| Holmberg, et al.^4^ | 1995 | Cancer Prevention Study I | United States | 384,796 | range: >=30 | 100 | up to: 13 | maternal age at birth |
| Hart and Smith^5^ | 2003 | Collaborative Study | United Kingdom | 5,765 | mean: 48.23 | 0 | up to: 25 | number of childbirths |
| Westergaard, et al.^6^ | 1997 | Danish Civil Registration System | Denmark | 2,092,128 | range: 0 to 15 | NR | mean: 14.86 | number of childbirths, birth order, maternal age at birth |
| Westergaard, et al.^7^ | 1997 | Danish Civil Registration System | Denmark | 1,975,584 | range: 0 to 14 | NR | up to: 14 | birth order, maternal age at birth |
| Westergaard, et al.^8^ | 1998 | Danish Civil Registration System | Denmark | 1,015,994 | range: 0 to 18 | 0 | mean: 15.73 | number of childbirths, birth order, maternal age at birth |
| Schuz, et al.^9^ | 2015 | Danish birth cohort | Denmark | 2,461,283 | from birth | 48.7 | up to: 20 | birth order |
| Momen, et al.^10^ | 2014 | Denmark, Finland, Sweden birth registry | Denmark, Finland, Sweden | 7,029,843 | from birth | 48.7 | up to: 14 | delivery method |
| Olesen, et al.^11^ | 2009 | Denmark’s Civil Registration System (CRS) | Denmark | 2,594,783 | from birth | 48.6 | mean: 21.7 | number of childbirths, birth order, maternal age at birth |
| Ramlau-Hansen, et al.^12^ | 2009 | Denmark’s Civil Registration System (CRS) | Denmark | 1,333,873 | from birth | 0 | mean: 21.74 | birth order |
| Zhang, et al.^13^ | 1995 | Framingham Heart Study | United States | 2,662 | range: 29 to 6 | 100 | mean: 28.26 | maternal age at birth |
| Zhang, et al.^14^ | 1999 | Framingham Study | United States | 2,164 | mean: 43.63 | 0 | up to: 42 | maternal age at birth |
| Levine, et al.^15^ | 2017 | Israeli Jewish | Israel | 1,056,058 | mean: 17.3 | 0 | up to: 6 | number of childbirths, birth order, maternal age at birth |
| Maule, et al.^16^ | 2007 | Italian National Institute of Statistics (ISTAT) | Italy | 633,155 | from birth | NR | up to: 5 | maternal age at birth |
| Heuch, et al.^17^ | 1996 | Medical Birth Registry | Norway | 1,489,297 | From birth | NR | Mean: 11.2 | birth order, maternal age at birth |
| Heuch et al.^18^ | 1998 | Medical Birth Registry | Norway | 1,489,297 | From birth | NR | Mean: 11.6 | Birth order, maternal age at birth |
| Yang, et al.^19^ | 2019 | Million Women Study | United Kingdom | 548,741 | mean: 59.73 | 100 | Mean: 12.7 | Breastfeeding duration |
| Yip, et al.^20^ | 2006 | Multi-Generation Register (MGR) | Sweden | 4,300,000 | from birth | NR | up to: 15 | maternal age at birth |
| Murray, et al.^21^ | 2002 | Northern Ireland Child Health System | Ireland | 434,933 | from birth | 48.48 | up to: 15 | maternal age at birth |
| Michels, et al.^22^ | 2001 | Nurses' Health Study (NHS) | United States | 50,586 | mean: 61.34 | 100 | up to: 5 | breastfeeding duration |
| Xue, et al.^23^ | 2008 | Nurses’ Health Study (NHS) | United States | 74,757 | range: 30 to 5 | 100 | up to: 28 | breastfeeding duration |
| Xue, et al.^24^ | 2007 | Nurses’ Health Study (NHS) | United States | 109,773 | range: 30 to 5 | 100 | mean: 23.5 | maternal age at birth |
| Michels, et al.^22^ | 2001 | Nurses’ Health Study II (NHS II) | United States | 89,385 | mean: 36 | 100 | up to: 6 | breastfeeding duration |
| Dixon et al.^25^ | 2018 | Perinatal Data Collection (PDC) of New South Wales | Australia | 1,072,957 | From birth | 48.61 | Mean: 6.3 | Delivery method |
| Black, et al.^26^ | 2015 | Scottish Morbidity Record (SMR02) | United Kingdom | 321,287 | from birth | 48.83 | mean: 14.8 | delivery method |
| Imterat, et al.^27^ | 2018 | Soroka University Medical Center (SUMC) | Israel | 201,738 | from birth | 49.1 | up to: 18 | birth order, maternal age at birth |
| Greenbaum, et al.^28^ | 2018 | Soroka University Medical Center (SUMC) | Israel | 132,054 | from birth | 50.18 | up to: 15 | delivery method |
| Nilsen, et al.^29^ | 2005 | St Olav’s University Hospital | Norway | 35,697 | range: >=20 | 44.87 | median: 31 | birth order, maternal age at birth |
| Crump, et al.^30^ | 2014 | Swedish Birth Registry | Sweden | 3,571,574 | from birth | 48.6 | up to: 37 | birth order, maternal age at birth |
| Crump, et al.^31^ | 2012 | Swedish Birth Registry | Sweden | 3,571,574 | from birth | 48.6 | mean: 18.6 | birth order, maternal age at birth |
| Crump, et al.^32^ | 2015 | Swedish Birth Registry | Sweden | 3,571,574 | from birth | 48.6 | up to: 38 | birth order, maternal age at birth |
| Bevier, et al.^33^ | 2011 | Swedish Family-Cancer Database | Sweden | 5,657,455 | range: 0 to 29 | NR | up to: 45 | number of childbirths, birth order |
| Altieri and Hemminki^34^ | 2007 | Swedish Family-Cancer Database | Sweden | 11,000,000 | NR | NR | up to: 46 | number of childbirths, birth order |
| Altieri, et al.^35^ | 2006 | Swedish Family-Cancer Database | Sweden | 10,500,000 | range: 0 to 27 | NR | up to: 44 | number of childbirths, birth order |
| Altieri, et al.^36^ | 2006 | Swedish Family-Cancer Database | Sweden | 10,500,000 | range: 0 to 27 | NR | up to: 45 | number of childbirths, birth order |
| Hemminki, et al.^37^ | 1999 | Swedish Family-Cancer Database (Seond Generation Register) | Sweden | 6,000,000 | from birth | NR | up to: 15 | maternal age at birth |
| Hemminki and Kyyronen^38^ | 1999 | Swedish Family-Cancer Database (Seond Generation Register) | Sweden | 3,800,000 | range: 0 to 15 | NR | mean: 22 | maternal age at birth |
| Mogren, et al.^39^ | 1999 | Swedish Medical Birth Register | Sweden | 248,701 | from birth | NR | mean: 21.33 | birth order, maternal age at birth |
| Crump, et al.^40^ | 2012 | Swedish Medical Birth Registry | Sweden | 354,860 | from birth | 0 | mean: 31.56 | birth order, maternal age at birth |
| Petridou, et al.^41^ | 2015 | Swedish Medical Birth Registry | Sweden | 3,444,136 | from birth | 48.61 | median: 15 | birth order, maternal age at birth |
| Baranowska-Rataj, et al.^42^ | 2017 | Swedish multigenerational registers | Sweden | 2,638,898 | Mean: 40 | 48.68 | up to: 34 | Number of childbirths |
| Cha, et al.^43^ | 2011 | National birth and death registration databases of Statistics Korea | Korea | 6,479,406 | from birth | NR | mean: 6.61 | birth order, maternal age at birth |
