# Supplementary material for: Impact of maternal reproductive factors on cancer risks of offspring: A systematic review and meta-analysis of cohort studies
Source: PLoS One. 2020 Mar 30;15(3):e0230721. doi: 10.1371/journal.pone.0230721 (PMC7105118; doi:10.1371/journal.pone.0230721)
Supplement: S5 Table — (DOCX) [file pone.0230721.s005.docx]

**S5 Table. Risk of bias assessment of included studies**

| **Authors** | **Year** | **Cohort (s)** | **Study participation** | **Study attrition** | **Reproductive factor measurement** | **Outcome measurement** | **Study confounding** | **Overall risk of bias** |
| --- | --- | --- | --- | --- | --- | --- | --- | --- |
| **Overall cancer incidence** | | | | | | | | |
| Martin, et al. | 2005 | Boyd Orr Cohort | Low | Moderate | Low | Low | Moderate | Low |
| Schuz, et al. | 2015 | Danish birth cohort | Low | Low | Low | Low | High | Low |
| Momen, et al. | 2014 | Denmark, Finland, Sweden birth registry | Low | Moderate | Low | Low | High | Low |
| Dixon, et al. | 2018 | Perinatal Data Collection (PDC) of New South Wales | Low | Moderate | Lo | Low | High | Low |
| Black, et al. | 2015 | Scottish Morbidity Record (SMR02) | Low | Moderate | Low | Low | High | Low |
| Imterat, et al. | 2018 | Soroka University Medical Center (SUMC) | Low | Low | Low | Low | High | Low |
| Bevier, et al. | 2011 | Swedish Family-Cancer Database | Low | Moderate | Low | Low | Moderate | Low |
| Mogren, et al. | 1999 | Swedish Medical Birth Register | Low | Moderate | Low | Low | High | Low |
| **Esophagus cancer incidence** | | | | | | | | |
| Yang, et al. | 2019 | Million Women Study | Low | Moderate | High | Low | High | High |
| Bevier, et al. | 2011 | Swedish Family-Cancer Database | Low | Moderate | Low | Low | Moderate | Low |
| **Stomach cancer incidence** | | | | | | | | |
| Martin, et al. | 2005 | Boyd Orr Cohort | Low | Moderate | Low | Low | Moderate | Low |
| Yang, et al. | 2019 | Million Women Study | Low | Moderate | High | Low | High | High |
| Altieri and Hemminki | 2007 | Swedish Family-Cancer Database | Low | Moderate | Low | Low | High | Low |
| Bevier, et al. | 2011 | Swedish Family-Cancer Database | Low | Moderate | Low | Low | Moderate | Low |
| **Colorectum cancer incidence** | | | | | | | | |
| Martin, et al. | 2005 | Boyd Orr Cohort | Low | Moderate | Low | Low | Moderate | Low |
| Yang, et al. | 2019 | Million Women Study | Low | Moderate | High | Low | High | High |
| Nilsen, et al. | 2005 | St Olav’s University Hospital | Low | Moderate | Low | Low | High | Low |
| Nilsen, et al. | 2005 | St Olav’s University Hospital | Low | Moderate | Low | Low | High | Low |
| Altieri and Hemminki | 2007 | Swedish Family-Cancer Database | Low | Moderate | Low | Low | High | Low |
| Bevier, et al. | 2011 | Swedish Family-Cancer Database | Low | Moderate | Low | Low | Moderate | Low |
| Hemminki and Kyyronen | 1999 | Swedish Family-Cancer Database (Seond Generation Register) | Low | Moderate | Low | Low | High | Low |
| **Liver cancer incidence** | | | | | | | | |
| Schuz, et al. | 2015 | Danish birth cohort | Low | Low | Low | Low | High | Low |
| Momen, et al. | 2014 | Denmark, Finland, Sweden birth registry | Low | Moderate | Low | Low | High | Low |
| Altieri and Hemminki | 2007 | Swedish Family-Cancer Database | Low | Moderate | Low | Low | High | Low |
| Bevier, et al. | 2011 | Swedish Family-Cancer Database | Low | Moderate | Low | Low | Moderate | Low |
| **Pancreas cancer incidence** | | | | | | | | |
| Yang, et al. | 2019 | Million Women Study | Low | Moderate | High | Low | High | High |
| Altieri and Hemminki | 2007 | Swedish Family-Cancer Database | Low | Moderate | Low | Low | High | Low |
| Bevier, et al. | 2011 | Swedish Family-Cancer Database | Low | Moderate | Low | Low | Moderate | Low |
| **Larynx cancer incidence** | | | | | | | | |
| Bevier, et al. | 2011 | Swedish Family-Cancer Database | Low | Moderate | Low | Low | Moderate | Low |
| **Lung cancer incidence** | | | | | | | | |
| Yang, et al. | 2019 | Million Women Study | Low | Moderate | High | Low | High | High |
| Altieri and Hemminki | 2007 | Swedish Family-Cancer Database | Low | Moderate | Low | Low | High | Low |
| Bevier, et al. | 2011 | Swedish Family-Cancer Database | Low | Moderate | Low | Low | Moderate | Low |
| Hemminki and Kyyronen | 1999 | Swedish Family-Cancer Database (Seond Generation Register) | Low | Moderate | Low | Low | High | Low |
| **Melanoma incidence** | | | | | | | | |
| Olesen, et al. | 2009 | Denmark’s Civil Registration System (CRS) | Low | Moderate | Low | Low | Moderate | Low |
| Yang, et al. | 2019 | Million Women Study | Low | Moderate | High | Low | High | High |
| Crump, et al. | 2014 | Swedish Birth Registry | Low | Moderate | Low | Low | Moderate | Low |
| Altieri and Hemminki | 2007 | Swedish Family-Cancer Database | Low | Moderate | Low | Low | High | Low |
| Bevier, et al. | 2011 | Swedish Family-Cancer Database | Low | Moderate | Low | Low | Moderate | Low |
| Hemminki and Kyyronen | 1999 | Swedish Family-Cancer Database (Seond Generation Register) | Low | Moderate | Low | Low | High | Low |
| **Breast cancer incidence** | | | | | | | | |
| Barber, et al. | 2019 | Black Women’s Health Study (BWHS) | Low | Low | High | Low | High | High |
| Martin, et al. | 2005 | Boyd Orr Cohort | Low | Moderate | Low | Low | Moderate | Low |
| Zhang, et al. | 1995 | Framingham Heart Study | Low | Low | High | Moderate | High | High |
| Yang, et al. | 2019 | Million Women Study | Low | Moderate | High | Low | High | High |
| Michels, et al. | 2001 | Nurses' Health Study (NHS) | Low | Low | High | Moderate | High | High |
| Michels, et al. | 2001 | Nurses' Health Study II (NHS II) | Low | Low | High | Moderate | High | High |
| Xue, et al. | 2007 | Nurses’ Health Study (NHS) | Low | High | High | Moderate | High | High |
| Altieri and Hemminki | 2007 | Swedish Family-Cancer Database | Low | Moderate | Low | Low | High | Low |
| Bevier, et al. | 2011 | Swedish Family-Cancer Database | Low | Moderate | Low | Low | Moderate | Low |
| Hemminki and Kyyronen | 1999 | Swedish Family-Cancer Database (Seond Generation Register) | Low | Moderate | Low | Low | High | Low |
| Mogren, et al. | 1999 | Swedish Medical Birth Register | Low | Moderate | Low | Low | High | Low |
| **Cervix uteri cancer incidence** | | | | | | | | |
| Altieri and Hemminki | 2007 | Swedish Family-Cancer Database | Low | Moderate | Low | Low | High | Low |
| Bevier, et al. | 2011 | Swedish Family-Cancer Database | Low | Moderate | Low | Low | Moderate | Low |
| Hemminki and Kyyronen | 1999 | Swedish Family-Cancer Database (Seond Generation Register) | Low | Moderate | Low | Low | High | Low |
| Mogren, et al. | 1999 | Swedish Medical Birth Register | Low | Moderate | Low | Low | High | Low |
| **Corpus uteri cancer incidence** | | | | | | | | |
| Yang, et al. | 2019 | Million Women Study | Low | Moderate | High | Low | High | High |
| Xue, et al. | 2008 | Nurses’ Health Study | Low | Low | High | Moderate | Moderate | Low |
| Altieri and Hemminki | 2007 | Swedish Family-Cancer Database | Low | Moderate | Low | Low | High | Low |
| Bevier, et al. | 2011 | Swedish Family-Cancer Database | Low | Moderate | Low | Low | Moderate | Low |
| Hemminki and Kyyronen | 1999 | Swedish Family-Cancer Database (Seond Generation Register) | Low | Moderate | Low | Low | High | Low |
| **Ovary cancer incidence** | | | | | | | | |
| Yang, et al. | 2019 | Million Women Study | Low | Moderate | High | Low | High | High |
| Altieri and Hemminki | 2007 | Swedish Family-Cancer Database | Low | Moderate | Low | Low | High | Low |
| Bevier, et al. | 2011 | Swedish Family-Cancer Database | Low | Moderate | Low | Low | Moderate | Low |
| **Prostate cancer incidence** | | | | | | | | |
| Martin, et al. | 2005 | Boyd Orr Cohort | Low | Moderate | Low | Low | Moderate | Low |
| Zhang, et al. | 1999 | Framingham Study | Low | Moderate | High | Moderate | High | High |
| Altieri and Hemminki | 2007 | Swedish Family-Cancer Database | Low | Moderate | Low | Low | High | Low |
| Bevier, et al. | 2011 | Swedish Family-Cancer Database | Low | Moderate | Low | Low | Moderate | Low |
| Hemminki and Kyyronen | 1999 | Swedish Family-Cancer Database (Seond Generation Register) | Low | Moderate | Low | Low | High | Low |
| **Testis cancer incidence** | | | | | | | | |
| Westergaard, et al. | 1998 | Danish Civil Registration System | Low | Low | Low | Low | High | Low |
| Momen, et al. | 2014 | Denmark, Finland, Sweden birth registry | Low | Moderate | Low | Low | High | Low |
| Ramlau-Hansen, et al. | 2009 | Denmark’s Civil Registration System (CRS) | Low | Low | Low | Low | High | Low |
| Levine, et al. | 2017 | Israeli Jewish | Low | Moderate | High | Low | High | High |
| Altieri and Hemminki | 2007 | Swedish Family-Cancer Database | Low | Moderate | Low | Low | High | Low |
| Bevier, et al. | 2011 | Swedish Family-Cancer Database | Low | Moderate | Low | Low | Moderate | Low |
| Hemminki and Kyyronen | 1999 | Swedish Family-Cancer Database (Seond Generation Register) | Low | Moderate | Low | Low | High | Low |
| Mogren, et al. | 1999 | Swedish Medical Birth Register | Low | Moderate | Low | Low | High | Low |
| Crump, et al. | 2012 | Swedish Medical Birth Registry | Low | Moderate | Low | Low | Moderate | Low |
| **Kidney cancer incidence** | | | | | | | | |
| Schuz, et al. | 2015 | Danish birth cohort | Low | Low | Low | Low | High | Low |
| Momen, et al. | 2014 | Denmark, Finland, Sweden birth registry | Low | Moderate | Low | Low | High | Low |
| Heuch, et al. | 1996 | Medical Birth Registry of Norway | Low | Moderate | Low | Low | High | Low |
| Yip, et al. | 2006 | Multi-Generation Register (MGR) | Low | Moderate | Low | Low | High | Low |
| Altieri and Hemminki | 2007 | Swedish Family-Cancer Database | Low | Moderate | Low | Low | High | Low |
| Bevier, et al. | 2011 | Swedish Family-Cancer Database | Low | Moderate | Low | Low | Moderate | Low |
| Hemminki and Kyyronen | 1999 | Swedish Family-Cancer Database (Seond Generation Register) | Low | Moderate | Low | Low | High | Low |
| Mogren, et al. | 1999 | Swedish Medical Birth Register | Low | Moderate | Low | Low | High | Low |
| **Bladder cancer incidence** | | | | | | | | |
| Altieri and Hemminki | 2007 | Swedish Family-Cancer Database | Low | Moderate | Low | Low | High | Low |
| Bevier, et al. | 2011 | Swedish Family-Cancer Database | Low | Moderate | Low | Low | Moderate | Low |
| **Thyroid cancer incidence** | | | | | | | | |
| Altieri and Hemminki | 2007 | Swedish Family-Cancer Database | Low | Moderate | Low | Low | High | Low |
| Bevier, et al. | 2011 | Swedish Family-Cancer Database | Low | Moderate | Low | Low | Moderate | Low |
| Hemminki and Kyyronen | 1999 | Swedish Family-Cancer Database (Seond Generation Register) | Low | Moderate | Low | Low | High | Low |
| **Brain and CNS cancer incidence** | | | | | | | | |
| Schuz, et al. | 2015 | Danish birth cohort | Low | Low | Low | Low | High | Low |
| Heuch, et al. | 1998 | Medical Birth Registry of Norway | Low | Moderate | Low | Low | High | Low |
| Yip, et al. | 2006 | Multi-Generation Register (MGR) | Low | Moderate | Low | Low | High | Low |
| Imterat, et al. | 2018 | Soroka University Medical Center (SUMC) | Low | Low | Low | Low | High | Low |
| Crump, et al. | 2015 | Swedish Birth Registry | Low | Moderate | Low | Low | Moderate | Low |
| Altieri, et al. | 2006 | Swedish Family-Cancer Database | Low | Moderate | Low | Low | Moderate | Low |
| Bevier, et al. | 2011 | Swedish Family-Cancer Database | Low | Moderate | Low | Low | Moderate | Low |
| Hemminki, et al. | 1999 | Swedish Family-Cancer Database (Seond Generation Register) | Low | Moderate | Low | Low | High | Low |
| Hemminki and Kyyronen | 1999 | Swedish Family-Cancer Database (Seond Generation Register) | Low | Moderate | Low | Low | High | Low |
| **Multiple myeloma incidence** | | | | | | | | |
| Lu, et al. | 2010 | California Teachers Study | Low | Moderate | High | Low | Low | Low |
| Altieri, et al. | 2006 | Swedish Family-Cancer Database | Low | Moderate | Low | Low | Moderate | Low |
| Bevier, et al. | 2011 | Swedish Family-Cancer Database | Low | Moderate | Low | Low | Moderate | Low |
| **Leukemia incidence** | | | | | | | | |
| Lu, et al. | 2010 | California Teachers Study | Low | Moderate | High | Low | Low | Low |
| Westergaard, et al. | 1997 | Danish Civil Registration System | Low | Low | Low | Low | High | Low |
| Schuz, et al. | 2015 | Danish birth cohort | Low | Low | Low | Low | High | Low |
| Momen, et al. | 2014 | Denmark, Finland, Sweden birth registry | Low | Moderate | Low | Low | High | Low |
| Maule, et al. | 2007 | Italian National Institute of Statistics (ISTAT) | Low | Moderate | Low | Low | High | Low |
| Yip, et al. | 2006 | Multi-Generation Register (MGR) | Low | Moderate | Low | Low | High | Low |
| Murray, et al. | 2002 | Northern Ireland Child Health System | Low | Moderate | Low | Low | High | Low |
| Imterat, et al. | 2018 | Soroka University Medical Center (SUMC) | Low | Low | Low | Low | High | Low |
| Greenbaum, et al. | 2018 | Soroka University Medical Center (SUMC) | Low | Low | Low | Low | High | Low |
| Altieri, et al. | 2006 | Swedish Family-Cancer Database | Low | Moderate | Low | Low | Moderate | Low |
| Bevier, et al. | 2011 | Swedish Family-Cancer Database | Low | Moderate | Low | Low | Moderate | Low |
| Hemminki, et al. | 1999 | Swedish Family-Cancer Database (Seond Generation Register) | Low | Moderate | Low | Low | High | Low |
| Hemminki and Kyyronen | 1999 | Swedish Family-Cancer Database (Seond Generation Register) | Low | Moderate | Low | Low | High | Low |
| Mogren, et al. | 1999 | Swedish Medical Birth Register | Low | Moderate | Low | Low | High | Low |
| **Lymphoma incidence** | | | | | | | | |
| Lu, et al. | 2010 | California Teachers Study | Low | Moderate | High | Low | Low | Low |
| Westergaard, et al. | 1997 | Danish Civil Registration System | Low | Low | Low | Low | High | Low |
| Schuz, et al. | 2015 | Danish birth cohort | Low | Low | Low | Low | High | Low |
| Momen, et al. | 2014 | Denmark, Finland, Sweden birth registry | Low | Moderate | Low | Low | High | Low |
| Yang, et al. | 2019 | Million Women Study | Low | Moderate | High | Low | High | High |
| Yip, et al. | 2006 | Multi-Generation Register (MGR) | Low | Moderate | Low | Low | High | Low |
| Imterat, et al. | 2018 | Soroka University Medical Center (SUMC) | Low | Low | Low | Low | High | Low |
| Crump, et al. | 2012 | Swedish Birth Registry | Low | Moderate | Low | Low | Moderate | Low |
| Altieri, et al. | 2006 | Swedish Family-Cancer Database | Low | Moderate | Low | Low | Moderate | Low |
| Bevier, et al. | 2011 | Swedish Family-Cancer Database | Low | Moderate | Low | Low | Moderate | Low |
| Hemminki and Kyyronen | 1999 | Swedish Family-Cancer Database (Seond Generation Register) | Low | Moderate | Low | Low | High | Low |
| Petridou, et al. | 2015 | Swedish Medical Birth Registry | Low | Moderate | Low | Low | High | Low |
| **Eye cancer incidence** | | | | | | | | |
| Schuz, et al. | 2015 | Danish birth cohort | Low | Low | Low | Low | High | Low |
| Momen, et al. | 2014 | Denmark, Finland, Sweden birth registry | Low | Moderate | Low | Low | High | Low |
| Yip, et al. | 2006 | Multi-Generation Register (MGR) | Low | Moderate | Low | Low | High | Low |
| Bevier, et al. | 2011 | Swedish Family-Cancer Database | Low | Moderate | Low | Low | Moderate | Low |
| **Bone cancer incidence** | | | | | | | | |
| Schuz, et al. | 2015 | Danish birth cohort | Low | Low | Low | Low | High | Low |
| Momen, et al. | 2014 | Denmark, Finland, Sweden birth registry | Low | Moderate | Low | Low | High | Low |
| Altieri and Hemminki | 2007 | Swedish Family-Cancer Database | Low | Moderate | Low | Low | High | Low |
| Bevier, et al. | 2011 | Swedish Family-Cancer Database | Low | Moderate | Low | Low | Moderate | Low |
| **Connective and soft tissue cancer incidence** | | | | | | | | |
| Schuz, et al. | 2015 | Danish birth cohort | Low | Low | Low | Low | High | Low |
| Altieri and Hemminki | 2007 | Swedish Family-Cancer Database | Low | Moderate | Low | Low | High | Low |
| Bevier, et al. | 2011 | Swedish Family-Cancer Database | Low | Moderate | Low | Low | Moderate | Low |
| **Overall cancer mortality** | | | | | | | | |
| Martin, et al. | 2005 | Boyd Orr Cohort | Low | Moderate | Low | Low | Moderate | Low |
| Cha, et al. | 2011 | national birth and death registration databases of Statistics Korea | Low | Moderate | Low | Low | High | Low |
| Baranowska-Rataj, et al. | 2017 | Swedish multigenerational registers | Low | Moderate | Low | Moderate | High | Low |
| **Stomach cancer mortality** | | | | | | | | |
| Martin, et al. | 2005 | Boyd Orr Cohort | Low | Moderate | Low | Low | Moderate | Low |
| Hart and Smith | 2003 | Collaborative Study | Low | Moderate | High | Low | High | High |
| **Colorectum cancer mortality** | | | | | | | | |
| Martin, et al. | 2005 | Boyd Orr Cohort | Low | Moderate | Low | Low | Moderate | Low |
| **Liver cancer mortality** | | | | | | | | |
| Cha, et al. | 2011 | national birth and death registration databases of Statistics Korea | Low | Moderate | Low | Low | High | Low |
| **Lung cancer mortality** | | | | | | | | |
| Hart and Smith | 2003 | Collaborative Study | Low | Moderate | High | Low | High | High |
| **Breast cancer mortality** | | | | | | | | |
| Martin, et al. | 2005 | Boyd Orr Cohort | Low | Moderate | Low | Low | Moderate | Low |
| Holmberg, et al. | 1995 | Cancer Prevention Study I | Moderate | Low | High | Moderate | High | High |
| **Prostate cancer mortality** | | | | | | | | |
| Martin, et al. | 2005 | Boyd Orr Cohort | Low | Moderate | Low | Low | Moderate | Low |
| **Brain and CNS cancer mortality** | | | | | | | | |
| Cha, et al. | 2011 | National birth and death registration databases of Statistics Korea | Low | Moderate | Low | Low | High | Low |
| **Leukemia mortality** | | | | | | | | |
| Cha, et al. | 2011 | National birth and death registration databases of Statistics Korea | Low | Moderate | Low | Low | High | Low |
| **Lymphoma mortality** | | | | | | | | |
| Cha, et al. | 2011 | National birth and death registration databases of Statistics Korea | Low | Moderate | Low | Low | High | Low |
| **Eye cancer mortality** | | | | | | | | |
| Cha, et al. | 2011 | National birth and death registration databases of Statistics Korea | Low | Moderate | Low | Low | High | Low |
| **Bone cancer mortality** | | | | | | | | |
| Cha, et al. | 2011 | National birth and death registration databases of Statistics Korea | Low | Moderate | Low | Low | High | Low |
| **Connective and soft tissue cancer mortality** | | | | | | | | |
| Cha, et al. | 2011 | National birth and death registration databases of Statistics Korea | Low | Moderate | Low | Low | High | Low |
