# Supplementary material for: Impact of maternal reproductive factors on cancer risks of offspring: A systematic review and meta-analysis of cohort studies
Source: PLoS One. 2020 Mar 30;15(3):e0230721. doi: 10.1371/journal.pone.0230721 (PMC7105118; doi:10.1371/journal.pone.0230721)
Supplement: S6 Table — (DOCX) [file pone.0230721.s006.docx]

**S6 Table. Summary of finding for maternal reproductive factors and childhood cancer incidence and mortality**

| **Outcomes (no of studies)** | **No of cases/participants, follow-up years** | **Relative risk (95% CI)** | **Population risk**  **(per 10,000) ^[[1]](#endnote-1)^** | **Risk difference**  **(per 10,000)** | **Certainty of the evidence** | **Plain language summary** |
| --- | --- | --- | --- | --- | --- | --- |
| **Higher maternal age at childbirth compared to 25 to 29 maternal age** | | | | | | |
| Overall cancer incidence (1) | 314/175593, Up to 18 | 0.73 (0.36-1.46) | 30 | 8 fewer (19 fewer to 14 more) | VERY LOW (due to observational design, imprecision)^[[2]](#endnote-2)^ | We are uncertain of the effects of higher maternal age at birth on overall cancer incidence |
| Kidney cancer incidence (2) | 211/NR, Up to 15 | 0.86 (0.47-1.56) | 1 | 0 fewer (1 fewer to 1 more) | LOW (due to observational design) | Higher maternal age at birth may have little or no effect on kidney cancer incidence |
| Brain and CNS cancer incidence (4) | 1239/>175,593, Up to 18 | 0.91 (0.73-1.14) | 4 | 1 fewer (1 fewer to 1 more) | LOW (due to observational design) | Higher maternal age at birth may have little or no effect on brain and CNS cancer incidence |
| Leukemia incidence (6) | 1613/>609,909, Up to 18 | 1.20 (0.95-1.50) | 8 | 2 more (0 fewer to 4 more) | LOW (due to observational design) | Higher maternal age at birth may have little or no effect on leukemia incidence |
| Lymphoma incidence (4) | 644/>3,500,214, Up to 18 | 1.19 (0.93-1.53) | 3 | 1 more (0 fewer to 2 more) | LOW (due to observational design) | Higher maternal age at birth may have little or no effect on lymphoma incidence |
| Eye cancer incidence (1) | 97/NR, Up to 15 | 1.72 (0.69-4.33) | 1 | 1 more (0 fewer to 3 more) | LOW (due to observational design) | Higher maternal age at birth may have little or no effect on eye cancer incidence |
| Overall cancer mortality (1) | 1,078/NR, mean: 6.6 | 1.03 (0.90-1.70) | 6 | 0 fewer (1 fewer to 4 more) | LOW (due to observational design) | Higher maternal age at birth may have little or no effect on overall cancer mortality |
| Liver cancer mortality (1) | 38/NR, Mean 6.6 | 0.80 (0.38-1.68) | 1 | 0 fewer (1 fewer to 1 more) | LOW (due to observational design) | Higher maternal age at birth may have little or no effect on liver cancer mortality |
| Brain and CNS cancer mortality (1) | 250/NR, Mean 6.6 | 1.15 (0.88-1.50) | 2 | 0 fewer (0 fewer to 1 more) | LOW (due to observational design) | Higher maternal age at birth may have little or no effect on brain and CNS cancer mortality |
| Leukemia mortality (1) | 421/NR, Mean 6.6 | 0.94 (0.76-1.17) | 2 | 0 fewer (0 fewer to 0 fewer) | LOW (due to observational design) | Higher maternal age at birth may have little or no effect on leukemia mortality |
| Lymphoma mortality (1) | 44/NR, Mean 6.6 | 1.29 (0.70-2.38) | 1 | 0 fewer (0 fewer to 1 more) | LOW (due to observational design) | Higher maternal age at birth may have little or no effect on lymphoma mortality |
| Eye cancer mortality (1) | 29/NR, Mean 6.6 | 1.94 (0.93-4.07) | 1 | 1 more (0 fewer to 3 more) | LOW (due to observational design) | Higher maternal age at birth may have little or no effect on eye cancer mortality |
| Bone cancer mortality (1) | 25/NR, Mean 6.6 | 0.75 (0.30-1.92) | 1 | 0 fewer (1 fewer to 1 more) | LOW (due to observational design) | Higher maternal age at birth may have little or no effect on bone cancer mortality |
| Connective and soft tissue cancer mortality (1) | 39/NR, Mean 6.6 | 0.81 (0.39-1.69) | 1 | 0 fewer (1 fewer to 1 more) | LOW (due to observational design) | Higher maternal age at birth may have little or no effect on connective and soft tissue cancer mortality |
| **Lower maternal age at childbirth compared to 25 to 29 maternal age** | | | | | | |
| Kidney cancer incidence (2) | 282/NR, Up to 15 | 0.84 (0.64-1.11) | 1 | 0 fewer (0 fewer to 0 fewer) | LOW (due to observational design) | Lower maternal age at birth may have little or no effect on kidney cancer incidence |
| Brain and CNS cancer incidence (3) | 1971/NR, Up to 15 | 1.01 (0.92-1.10) | 4 | 0 fewer (0 fewer to 0 fewer) | LOW (due to observational design) | Lower maternal age at birth may have little or no effect on brain and CNS cancer incidence |
| Leukemia incidence (4) | 2092/>386,617, Up to 15 | 0.91 (0.82-1.01) | 8 | 1 fewer (1 fewer to 0 fewer) | LOW (due to observational design) | Lower maternal age at birth may have little or no effect on leukemia incidence |
| Lymphoma incidence (3) | 608/>3,015,865, Up to 15 | 1.19 (0.90-1.58) | 3 | 1 more (0 fewer to 2 more) | LOW (due to observational design) | Lower maternal age at birth may have little or no effect on lymphoma incidence |
| Eye cancer incidence (1) | 140/NR, Up to 15 | 0.71 (0.48-1.06) | 1 | 0 fewer (1 fewer to 0 fewer) | LOW (due to observational design) | Lower maternal age at birth may have little or no effect on eye cancer incidence |
| Liver cancer mortality (1) | 39/NR, Mean 6.6 | 0.83 (0.41-1.70) | 1 | 0 fewer (1 fewer to 1 more) | LOW (due to observational design) | Lower maternal age at birth may have little or no effect on overall cancer mortality |
| Brain and CNS cancer mortality (1) | 256/NR, Mean 6.6 | 1.28 (0.98-1.66) | 2 | 1 more (0 fewer to 1 more) | LOW (due to observational design) | Lower maternal age at birth may have little or no effect on brain and CNS cancer mortality |
| Leukemia mortality (1) | 454/NR, Mean 6.6 | 1.16 (0.95-1.42) | 2 | 0 fewer (0 fewer to 1 more) | LOW (due to observational design) | Lower maternal age at birth may have little or no effect on leukemia mortality |
| Lymphoma mortality (1) | 45/NR, Mean 6.6 | 1.45 (0.79-2.66) | 1 | 0 fewer (0 fewer to 2 more) | LOW (due to observational design) | Lower maternal age at birth may have little or no effect on lymphoma mortality |
| Eye cancer mortality (1) | 23/NR, Mean 6.6 | 1.00 (0.40-2.46) | 1 | 0 fewer (1 fewer to 1 more) | LOW (due to observational design) | Lower maternal age at birth may have little or no effect on eye cancer mortality |
| Bone cancer mortality (1) | 26/NR, Mean 6.6 | 1.02 (0.44-2.39) | 1 | 0 fewer (1 fewer to 1 more) | LOW (due to observational design) | Lower maternal age at birth may have little or no effect on bone cancer mortality |
| Connective and soft tissue cancer mortality (1) | 40/NR, Mean 6.6 | 0.77 (0.36-1.67) | 1 | 0 fewer (1 fewer to 1 more) | LOW (due to observational design) | Lower maternal age at birth may have little or no effect on connective and soft tissue cancer mortality |
| **Higher birth order compared to lower birth order** | | | | | | |
| Overall cancer incidence (2) | 3585/1621890, Up to 20 | 0.99 (0.90-1.10) | 30 | 0 fewer (3 fewer to 3 more) | LOW (due to observational design) | Higher birth order may have little or no effect on overall cancer incidence |
| Liver cancer incidence (1) | 34/1218414, Up to 20 | 1.02 (0.33-3.13) | 1 | 0 fewer (1 fewer to 2 more) | LOW (due to observational design) | Higher birth order may have little or no effect on liver cancer incidence |
| Testis cancer incidence (1) | 2151/NR, mean 21.7 | 1.00 (0.90-1.00) | 2 | 0 fewer (0 fewer to 0 fewer) | LOW (due to observational design) | Higher birth order may have little or no effect on testis cancer incidence |
| Kidney cancer incidence (2) | >122/>1,218,414, Up to 20 | 1.06 (0.75-1.51) | 1 | 0 fewer (0 fewer to 1 more) | LOW (due to observational design) | Higher birth order may have little or no effect on kidney cancer incidence |
| Brain and CNS cancer incidence (2) | 1048/>1218414, Up to 20 | 1.04 (0.87-1.24) | 4 | 0 fewer (1 fewer to 1 more) | LOW (due to observational design) | Higher birth order may have little or no effect on brain and CNS cancer incidence |
| Leukemia incidence (2) | 1,154/>1218414, Up to 20 | 0.75 (0.61-1.07) | 8 | 2 fewer (3 fewer to 1 more) | LOW (due to observational design) | Higher birth order may have little or no effect on leukemia incidence |
| Lymphoma incidence (2) | 482/>1218414, Up to 20 | 1.18 (0.82-1.69) | 3 | 1 more (1 fewer to 2 more) | LOW (due to observational design) | Higher birth order may have little or no effect on lymphoma incidence |
| Eye cancer incidence (1) | 70/1218414, Up to 20 | 1.48 (0.71-3.08) | 1 | 0 fewer (0 fewer to 2 more) | LOW (due to observational design) | Higher birth order may have little or no effect on eye cancer incidence |
| Bone cancer incidence (1) | 121/1218414, Up to 20 | 1.12 (0.60-2.09) | 2 | 0 fewer (1 fewer to 2 more) | LOW (due to observational design) | Higher birth order may have little or no effect on bone cancer incidence |
| Connective and soft tissue cancer incidence (1) | 161/1218414, Up to 20 | 0.79 (0.43-1.45) | 2 | 0 fewer (1 fewer to 1 more) | LOW (due to observational design) | Higher birth order may have little or no effect on connective and soft tissue cancer incidence |
| Overall cancer mortality (1) | 850/NR, Mean: 6.6 | 1.01 (0.84-1.22) | 6 | 0 fewer (1 fewer to 1 more) | LOW (due to observational design) | Higher birth order may have little or no effect on overall cancer mortality |
| Liver cancer mortality (1) | 36/NR, Mean: 6.6 | 0.61 (0.21-1.77) | 1 | 0 fewer (1 fewer to 1 more) | LOW (due to observational design) | Higher birth order may have little or no effect on liver cancer mortality |
| Brain and CNS cancer mortality (1) | 201/NR, Mean: 6.6 | 0.74 (0.49-1.12) | 2 | 1 fewer (1 fewer to 0 fewer) | LOW (due to observational design) | Higher birth order may have little or no effect on brain and CNS cancer mortality |
| Leukemia mortality (1) | 323/NR, Mean: 6.6 | 1.14 (0.86-1.53) | 2 | 0 fewer (0 fewer to 1 more) | LOW (due to observational design) | Higher birth order may have little or no effect on leukemia mortality |
| Lymphoma mortality (1) | 44/NR, Mean: 6.6 | 1.36 (0.66-2.80) | 1 | 0 fewer (0 fewer to 2 more) | LOW (due to observational design) | Higher birth order may have little or no effect on lymphoma mortality |
| Eye cancer mortality (1) | 17/NR, Mean: 6.6 | 2.57 (0.89-7.44) | 1 | 2 more (0 fewer to 6 more) | LOW (due to observational design) | Higher birth order may have little or no effect on eye cancer mortality |
| Bone cancer mortality (1) | 22/NR, Mean: 6.6 | 1.30 (0.43-3.91) | 1 | 0 fewer (1 fewer to 3 more) | LOW (due to observational design) | Higher birth order may have little or no effect on bone cancer mortality |
| Connective and soft tissue cancer mortality (1) | 30/NR, Mean: 6.6 | 0.69 (0.20-2.34) | 1 | 0 fewer (1 fewer to 1 more) | LOW (due to observational design) | Higher birth order may have little or no effect on connective and soft tissue cancer mortality |
| **Higher number of childbirths compared to smaller number of childbirths** | | | | | | |
| Testis cancer incidence (1) | 129/NR, Mean 15.7 | 0.81 (0.57-1.16) | 2 | 0 fewer (1 fewer to 0 fewer) | LOW (due to observational design) | Higher number of childbirths may have little or no effect on testis cancer incidence |
| **Cesarean delivery compared to vaginal delivery** | | | | | | |
| Overall cancer incidence (3) | 12,763/8,244,356, Up to 14.8 | 1.10 (0.99-1.23) | 30 | 3 more (0 fewer to 7 more) | LOW (due to observational design) | Cesarean delivery may have little or no effect on cancer incidence |
| Liver cancer incidence (1) | 136/6,907,253, Up to 14 | 1.27 (0.79-2.05) | 1 | 0 fewer (0 fewer to 1 more) | LOW (due to observational design) | Cesarean delivery may have little or no effect on liver cancer incidence |
| Testis cancer incidence (1) | 104/6,907,253, Up to 14 | 1.64 (0.96-2.80) | 2 | 1 more (0 fewer to 4 more) | LOW (due to observational design) | Cesarean delivery may have little or no effect on testis cancer incidence |
| Leukemia incidence (2) | 3546/7,039,307, Up to 15 | 1.62 (0.55-4.77) | 8 | 5 more (4 fewer to 30 more) | VERY LOW (due to observational design, inconsistency, imprecision)^[[3]](#endnote-3)^ | We are uncertain of the effects of Cesarean delivery on leukemia incidence |
| Lymphoma incidence (1) | 635/6,907,253, Up to 14 | 1.21 (0.96-1.52) | 3 | 1 more (0 fewer to 2 more) | LOW (due to observational design) | Cesarean delivery may have little or no effect on lymphoma incidence |
| Eye cancer incidence (1) | 416/6,907,253, Up to 14 | 0.86 (0.63-1.18) | 1 | 0 fewer (0 fewer to 0 fewer) | LOW (due to observational design) | Cesarean delivery may have little or no effect on eye cancer incidence |
| Bone cancer incidence (1) | 386/6,907,253, Up to 14 | 1.18 (0.85-1.63) | 2 | 0 fewer (0 fewer to 1 more) | LOW (due to observational design) | Cesarean delivery may have little or no effect on bone cancer incidence |

CI=Confidence Interval; NR, Not Reported

1. Cumulative risk between 0 to 19 years from the International Agency Research on Cancer online analysis system. [↑](#endnote-ref-1)
2. Confidence interval around absolute effect includes both appreciable benefit and harm. [↑](#endnote-ref-2)
3. I^2^=81.7%; Q-test p-value=0.019; Confidence interval around absolute effect includes both appreciable harm and no appreciable effect. [↑](#endnote-ref-3)
