# Supplementary material for: Impact of maternal reproductive factors on cancer risks of offspring: A systematic review and meta-analysis of cohort studies
Source: PLoS One. 2020 Mar 30;15(3):e0230721. doi: 10.1371/journal.pone.0230721 (PMC7105118; doi:10.1371/journal.pone.0230721)
Supplement: S7 Table — (DOCX) [file pone.0230721.s007.docx]

**S7 Table. Maternal reproductive factors and cancer incidence and mortality in childhood**

| **Type of cancer** | **Risk of bias** | **No of studies** | **No of participants** | **No of cases** | **Follow-up year** | **Random effect**  **RR (95% CI)** | **I^2^, %**  **(p-value)** | **Interaction p-value** |
| --- | --- | --- | --- | --- | --- | --- | --- | --- |
| **Higher maternal age at childbirth compared to 25 to 29 maternal age** | | | | | | | | |
| Overall cancer incidence | Low | 1 | 175,593 | 314 | Up to 18 | 0.73 (0.36-1.46) | NA | NA |
| Kidney cancer incidence | Low | 2 | NR | 211 | Up to 15 | 0.86 (0.47-1.56) | 0.0 (0.955) | NA |
| CNS incidence | Low | 4 | >175,593 | 1,239 | Up to 18 | 0.91 (0.73-1.14) | 0.0 (0.960) | NA |
| Leukemia incidence | Low | 6 | >609,909 | 1,613 | Up to 18 | 1.20 (0.95-1.50) | 0.0 (0.836) | NA |
| Lymphoma incidence | Low | 4 | >3,500,214 | 644 | Up to 18 | 1.19 (0.93-1.53) | 0.0 (0.907) | NA |
| Eye cancer incidence | Low | 1 | NR | 97 | Up to 15 | 1.72 (0.69-4.33) | NA | NA |
| Overall mortality | Low | 1 | NR | 1,078 | Mean 6.6 | 1.03 (0.90-1.17) | NA | NA |
| Liver mortality | Low | 1 | NR | 38 | Mean 6.6 | 0.80 (0.38-1.68) | NA | NA |
| CNS mortality | Low | 1 | NR | 250 | Mean 6.6 | 1.15 (0.88-1.50) | NA | NA |
| Leukemia mortality | Low | 1 | NR | 421 | Mean 6.6 | 0.94 (0.76-1.17) | NA | NA |
| Lymphoma mortality | Low | 1 | NR | 44 | Mean 6.6 | 1.29 (0.70-2.38) | NA | NA |
| Eye cancer mortality | Low | 1 | NR | 29 | Mean 6.6 | 1.94 (0.93-4.07) | NA | NA |
| Bone cancer mortality | Low | 1 | NR | 25 | Mean 6.6 | 0.75 (0.30-1.92) | NA | NA |
| Soft tissue cancer mortality | Low | 1 | NR | 39 | Mean 6.6 | 0.81 (0.39-1.69) | NA | NA |
| **Lower maternal age at childbirth compared to 25 to 29 maternal age** | | | | | | | | |
| Kidney cancer incidence | Low | 2 | NR | 282 | Up to 15 | 0.84 (0.64-1.11) | 0.0 (0.891) | NA |
| Brain and CNS cancer incidence | Low | 3 | NR | 1,971 | Up to 15 | 1.01 (0.92-1.10) | 0.0 (0.812) | NA |
| Leukemia incidence | Low | 4 | >386,617 | 2,092 | Up to 15 | 0.91 (0.82-1.01) | 0.01 (0.329) | NA |
| Lymphoma incidence | Low | 3 | >3,015,865 | 608 | Up to 15 | 1.19 (0.90-1.58) | 0.0 (0.627) | NA |
| Eye cancer incidence | Low | 1 | NR | 140 | Up to 15 | 0.71 (0.48-1.06) | NA | NA |
| Overall cancer mortality | Low | 1 | NR | 1,114 | Mean 6.6 | 1.15 (1.01-1.30) | NA | NA |
| Liver cancer mortality | Low | 1 | NR | 39 | Mean 6.6 | 0.83 (0.41-1.70) | NA | NA |
| CNS cancer mortality | Low | 1 | NR | 256 | Mean 6.6 | 1.28 (0.98-1.66) | NA | NA |
| Leukemia mortality | Low | 1 | NR | 454 | Mean 6.6 | 1.16 (0.95-1.42) | NA | NA |
| Lymphoma mortality | Low | 1 | NR | 45 | Mean 6.6 | 1.45 (0.79-2.66) | NA | NA |
| Eye cancer mortality | Low | 1 | NR | 23 | Mean 6.6 | 1.00 (0.40-2.46) | NA | NA |
| Bone cancer mortality | Low | 1 | NR | 26 | Mean 6.6 | 1.02 (0.44-2.39) | NA | NA |
| Soft tissue cancer mortality | Low | 1 | NR | 40 | Mean 6.6 | 0.77 (0.36-1.67) | NA | NA |
| **Higher birth order compared to lower birth order** | | | | | | | | |
| Overall cancer incidence | Low | 2 | 1,621,890 | 3,585 | Up to 20 | 0.99 (0.90-1.10) | 0.0 (0.495) | NA |
| Liver cancer incidence | Low | 1 | 1,218,414 | 34 | Up to 20 | 1.02 (0.33-3.13) | NA | NA |
| Testis cancer incidence | Low | 1 | NR | 2,151 | Mean 21.7 | 1.00 (0.90-1.00) | NA | NA |
| Kidney cancer incidence | Low | 2 | >1,218,414 | >122 | Up to 20 | 1.06 (0.75-1.51) | 0.0 (0.686) | NA |
| CNS cancer incidence | Low | 2 | >1,218,414 | 1,048 | Up to 20 | 1.04 (0.87-1.24) | 0.0 (0.834) | NA |
| Leukemia incidence | Low | 2 | >1,218,414 | 1,154 | Up to 20 | 0.75 (0.61-1.07) | 0.0 (0.428) | NA |
| Lymphoma incidence | Low | 2 | >1,218,414 | 482 | Up to 20 | 1.18 (0.82-1.69) | 0.0 (0.556) | NA |
| Eye cancer incidence | Low | 1 | 1,218,414 | 70 | Up to 20 | 1.48 (0.71-3.08) | NA | NA |
| Bone cancer incidence | Low | 1 | 1,218,414 | 121 | Up to 20 | 1.12 (0.60-2.09) | NA | NA |
| Connective and soft tissue cancer incidence | Low | 1 | 1,218,414 | 161 | Up to 20 | 0.79 (0.43-1.45) | NA | NA |
| Overall cancer mortality | Low | 1 | NR | 850 | Mean: 6.6 | 1.01 (0.84-1.22) | NA | NA |
| Liver cancer mortality | Low | 1 | NR | 36 | Mean: 6.6 | 0.61 (0.21-1.77) | NA | NA |
| CNS cancer mortality | Low | 1 | NR | 201 | Mean: 6.6 | 0.74 (0.49-1.12) | NA | NA |
| Leukemia mortality | Low | 1 | NR | 323 | Mean: 6.6 | 1.14 (0.86-1.53) | NA | NA |
| Lymphoma mortality | Low | 1 | NR | 44 | Mean: 6.6 | 1.36 (0.66-2.80) | NA | NA |
| Eye cancer mortality | Low | 1 | NR | 17 | Mean: 6.6 | 2.57 (0.89-7.44) | NA | NA |
| Bone cancer mortality | Low | 1 | NR | 22 | Mean: 6.6 | 1.30 (0.43-3.91) | NA | NA |
| Soft tissue cancer mortality | Low | 1 | NR | 30 | Mean: 6.6 | 0.69 (0.20-2.34) | NA | NA |
| **Higher number of childbirths compared to smaller number of childbirths** | | | | | | | | |
| Testis cancer incidence | Low | 1 | NR | 129 | Mean 15.7 | 0.81 (0.57-1.16) | NA | NA |
| CNS cancer incidence | Low | 1 | NR | 424 | Up to 14 | 1.27 (1.06-1.52) | NA | NA |
| Leukemia incidence | Low | 1 | NR | 306 | Up to 15 | 2.11 (1.62-2.75) | NA | NA |
| Lymphoma incidence | Low | 1 | NR | 13 | Mean 14.9 | 4.66 (1.40-15.57) | NA | NA |
| **Cesarean delivery compared to vaginal delivery** | | | | | | | | |
| Overall cancer incidence | Low | 3 | 8,244,356 | 12,763 | Up to 14.8 | 1.10 (0.99-1.23) | 53.3 (0.145) | NA |
| Liver cancer incidence | Low | 1 | 6,907,253 | 136 | Up to 14 | 1.27 (0.79-2.05) | NA | NA |
| Testis cancer incidence | Low | 1 | 6,907,253 | 104 | Up to 14 | 1.64 (0.96-2.80) | NA | NA |
| Kidney cancer incidence | Low | 1 | 6,907,253 | 717 | Up to 14 | 1.25 (1.01-1.55) | NA | NA |
| Leukemia incidence | Low | 2 | 7,039,307 | 3,546 | Up to 15 | 1.62 (0.55-4.77) | 81.7 (0.019) | NA |
| Lymphoma incidence | Low | 1 | 6,907,253 | 635 | Up to 14 | 1.21 (0.96-1.52) | NA | NA |
| Eye cancer incidence | Low | 1 | 6,907,253 | 416 | Up to 14 | 0.86 (0.63-1.18) | NA | NA |
| Bone cancer incidence | Low | 1 | 6,907,253 | 386 | Up to 14 | 1.18 (0.85-1.63) | NA | NA |

CI, confidence interval; NA, not applicable; NR, not reported; RR, relative risk
