# Supplementary material for: Impact of maternal reproductive factors on cancer risks of offspring: A systematic review and meta-analysis of cohort studies
Source: PLoS One. 2020 Mar 30;15(3):e0230721. doi: 10.1371/journal.pone.0230721 (PMC7105118; doi:10.1371/journal.pone.0230721)
Supplement: S8 Table — (DOCX) [file pone.0230721.s008.docx]

**S8 Table. Summary of finding for maternal reproductive factors and adult cancer incidence and mortality**

| **Outcomes (no of studies)** | **No of cases/participants, follow-up years** | **Relative risk (95% CI)** | **Population risk**  **(per 1,000) ^a^** | **Risk difference**  **(per 1,000)** | **Certainty of the evidence** | **Plain language summary** |
| --- | --- | --- | --- | --- | --- | --- |
| **Higher maternal age at childbirth compared to 25 to 29 maternal age** | | | | | | |
| Colorectum cancer incidence (1) | 118/16839, Median 31 | 0.83 (0.46-1.51) | 20 | 3 fewer (11 fewer to 10 more) | LOW (due to observational design) | Higher maternal age at birth may have little or no effect on colorectum cancer incidence |
| Breast cancer incidence (3) | 3156/>11,754, Up to 28 | 1.03 (0.88-1.20) | 90 | 3 more (11 fewer to 18 more) | VERY LOW (due to observational design, risk of bias, imprecision) ^b,c^ | We are uncertain of the effects of higher maternal age at birth on breast cancer incidence |
| Prostate cancer incidence (1) | 70/1089, Up to 42 | 1.10 (0.56-2.17) | 89 | 9 more (39 fewer to 104 more) | VERY LOW (due to observational design, risk of bias, imprecision) ^c,d^ | We are uncertain of the effects of higher maternal age at birth on prostate cancer incidence |
| Leukemia incidence (1) | 20/NR, Mean 11 | 1.19 (0.28-5.08) | 6 | 1 more (4 fewer to 24 more) | VERY LOW (due to observational design, imprecision) ^e^ | We are uncertain of the effects of higher maternal age at birth on leukemia incidence |
| Lymphoma incidence (1) | 215/NR, Mean 11 | 0.87 (0.49-1.54) | 10 | 1 fewer (5 fewer to 5 more) | LOW (due to observational design) | Higher maternal age at birth may have little or no effect on lymphoma incidence |
| Breast cancer mortality (1) | 555/106808, Up to 13 | 1.38 (0.87-2.21) | 15 | 6 more (2 fewer to 18 more) | VERY LOW (due to observational design, imprecision) ^e^ | We are uncertain of the effects of higher maternal age at birth on breast cancer mortality |
| **Lower maternal age at childbirth compared to 25 to 29 maternal age** | | | | | | |
| Colorectum cancer incidence (1) | 180/21325, Median 31 | 1.11 (0.82-1.49) | 20 | 2 more (4 fewer to 10 more) | LOW (due to observational design) | Lower maternal age at birth may have little or no effect on colorectal cancer incidence |
| Breast cancer incidence (3) | 3020/>14,498, Up to 28 | 0.93 (0.84-1.02) | 90 | 6 fewer (14 fewer to 2 more) | VERY LOW (due to observational design, risk of bias) ^b^ | We are uncertain of the effects of higher maternal age at birth on breast cancer incidence |
| Prostate cancer incidence (1) | 61/1009, Up to 42 | 1.00 (0.63-1.60) | 89 | 0 fewer (33 fewer to 53 more) | VERY LOW (due to observational design, risk of bias, imprecision) ^c,d^ | We are uncertain of the effects of higher maternal age at birth on prostate cancer incidence |
| Multiple myeloma incidence (1) | 68/NR, Mean 11 | 1.32 (0.75-2.32) | 4 | 1 more (1 fewer to 5 more) | LOW (due to observational design) | Lower maternal age at birth may have little or no effect on multiple myeloma incidence |
| Lymphoma incidence (1) | 339/NR, Mean 11 | 0.84 (0.65-1.08) | 10 | 2 fewer (4 fewer to 1 more) | LOW (due to observational design) | Lower maternal age at birth may have little or no effect on lymphoma incidence |
| Breast cancer mortality (1) | 655/128437, Up to 13 | 1.06 (0.87-1.30) | 15 | 1 more (2 fewer to 5 more) | LOW (due to observational design) | Lower maternal age at birth may have little or no effect on breast cancer mortality |
| **Higher birth order compared to lower birth order** | | | | | | |
| Overall cancer incidence (1) | 154854/11314910, Up to 45 | 0.88 (0.80-1.00) | 307 | 37 fewer (61 fewer to 0 fewer) | VERY LOW (due to observational design, imprecision) ^f^ | We are uncertain of the effects of higher birth order on overall cancer incidence |
| Esophagus cancer incidence (1) | 1366/11314910, Up to 45 | 0.70 (0.42-1.16) | 3 | 1 fewer (2 fewer to 0 fewer) | LOW (due to observational design) | Higher birth order may have little or no effect on esophagus cancer incidence |
| Gastric cancer incidence (1) | 2524/11314910, Up to 45 | 1.23 (0.86-1.69) | 7 | 2 more (1 fewer to 5 more) | LOW (due to observational design) | Higher birth order may have little or no effect on gastric cancer incidence |
| Colorectum cancer incidence (2) | 8658/11334451, Up to 45 | 0.90 (0.74-1.08) | 20 | 2 fewer (5 fewer to 2 more) | LOW (due to observational design) | Higher birth order may have little or no effect on colorectal cancer incidence |
| Liver cancer incidence (1) | 2548/11314910, Up to 45 | 0.78 (0.55-1.12) | 6 | 1 fewer (3 fewer to 1 more) | LOW (due to observational design) | Higher birth order may have little or no effect on liver cancer incidence |
| Pancreatic cancer incidence (1) | 3090/11314910, Up to 45 | 0.80 (0.53-1.23) | 8 | 2 fewer (4 fewer to 2 more) | LOW (due to observational design) | Higher birth order may have little or no effect on pancreatic cancer incidence |
| Larynx cancer incidence (1) | 760/11314910, Up to 45 | 1.16 (0.57-2.30) | 2 | 0 fewer (1 fewer to 3 more) | LOW (due to observational design) | Higher birth order may have little or no effect on larynx cancer incidence |
| Lung cancer incidence (1) | 11088/11314910, Up to 45 | 1.09 (0.91-1.30) | 33 | 3 more (3 fewer to 10 more) | LOW (due to observational design) | Higher birth order may have little or no effect on lung cancer incidence |
| Breast cancer incidence (2) | 29,844/>11,314,910, Up to 45 | 0.96 (0.88-1.06) | 90 | 4 fewer (11 fewer to 5 more) | LOW (due to observational design) | Higher birth order may have little or no effect on breast cancer incidence |
| Ovary cancer incidence (1) | 3966/11314910, Up to 45 | 1.09 (0.86-1.44) | 9 | 1 more (1 fewer to 4 more) | LOW (due to observational design) | Higher birth order may have little or no effect on ovary cancer incidence |
| Prostate cancer incidence (1) | 25072/11314910, Up to 45 | 1.09 (1.00-1.19) | 89 | 8 more (0 fewer to 17 more) | LOW (due to observational design, imprecision) ^e^ | We are uncertain of the effects of higher birth order on prostate cancer incidence |
| Testis cancer incidence (1) | 354/11314910, Up to 45 | 0.47 (0.22-1.06) | 4 | 2 fewer (3 fewer to 0 fewer) | LOW (due to observational design) | Higher birth order may have little or no effect on testis cancer incidence |
| Kidney cancer incidence (1) | 3800/11314910, Up to 45 | 0.97 (0.70-1.33) | 8 | 0 fewer (2 fewer to 3 more) | LOW (due to observational design) | Higher birth order may have little or no effect on kidney cancer incidence |
| Bladder cancer incidence (1) | 6092/11314910, Up to 45 | 0.83 (0.68-1.06) | 7 | 1 fewer (2 fewer to 0 fewer) | LOW (due to observational design) | Higher birth order may have little or no effect on bladder cancer incidence |
| Brain and CNS cancer incidence (1) | 5512/11314910, Up to 45 | 0.97 (0.78-1.23) | 4 | 0 fewer (1 fewer to 1 more) | LOW (due to observational design) | Higher birth order may have little or no effect on brain and CNS cancer incidence |
| Myeloma incidence (1) | 1734/11314910, Up to 45 | 1.12 (0.70-1.77) | 4 | 0 fewer (1 fewer to 3 more) | LOW (due to observational design) | Higher birth order may have little or no effect on myeloma incidence |
| Leukemia incidence (1) | 374/NR, Up to 45 | 1.01 (0.64-1.59) | 6 | 0 fewer (2 fewer to 4 more) | LOW (due to observational design) | Higher birth order may have little or no effect on leukemia incidence |
| Lymphoma incidence (1) | 329/NR, Up to 45 | 0.57 (0.21-1.58) | 10 | 4 fewer (8 fewer to 6 more) | LOW (due to observational design) | Higher birth order may have little or no effect on lymphoma incidence |
| Eye cancer incidence (1) | 468/11314910, Up to 45 | 0.73 (0.36-1.52) | 1 | 0 fewer (1 fewer to 1 more) | LOW (due to observational design) | Higher birth order may have little or no effect on eye cancer incidence |
| Bone cancer incidence (1) | 168/11314910, Up to 45 | 1.37 (0.33-5.74) | 1 | 0 fewer (1 fewer to 5 more) | LOW (due to observational design) | Higher birth order may have little or no effect on bone cancer incidence |
| Connective and soft tissue cancer incidence (1) | 830/11314910, Up to 45 | 1.00 (0.55-1.77) | 2 | 0 fewer (1 fewer to 2 more) | LOW (due to observational design) | Higher birth order may have little or no effect on connective and soft tissue cancer incidence |
| **Higher number of childbirths compared to smaller number of childbirths** | | | | | | |
| Overall cancer incidence (1) | 77427/5657455, Up to 45 | 0.97 (0.83-1.12) | 307 | 9 fewer (52 fewer to 37 more) | LOW (due to observational design, imprecision) ^c^ | We are uncertain of the effects of higher number of childbirths on overall cancer incidence |
| Esophagus cancer incidence (1) | 683/5657455, Up to 45 | 0.97 (0.55-1.64) | 3 | 0 fewer (1 fewer to 2 more) | LOW (due to observational design) | Higher number of childbirths may have little or no effect on esophagus cancer incidence |
| Colorectal cancer incidence (1) | 4255/5657455, Up to 45 | 0.86 (0.70-1.06) | 20 | 3 fewer (6 fewer to 1 more) | LOW (due to observational design) | Higher number of childbirths may have little or no effect on colorectal cancer incidence |
| Hepatic cancer incidence (1) | 1274/5657455, Up to 45 | 0.86 (0.57-1.26) | 6 | 1 fewer (3 fewer to 2 more) | LOW (due to observational design) | Higher number of childbirths may have little or no effect on hepatic cancer incidence |
| Pancreatic cancer incidence (1) | 1545/5657455, Up to 45 | 0.80 (0.53-1.19) | 8 | 2 fewer (4 fewer to 2 more) | LOW (due to observational design) | Higher number of childbirths may have little or no effect on pancreatic cancer incidence |
| Larynx cancer incidence (1) | 380/5657455, Up to 45 | 1.52 (0.75-3.05) | 2 | 1 more (1 fewer to 4 more) | LOW (due to observational design) | Higher number of childbirths may have little or no effect on larynx cancer incidence |
| Lung cancer incidence (1) | 5544/5657455, Up to 45 | 0.86 (0.70-1.06) | 33 | 5 fewer (10 fewer to 2 more) | LOW (due to observational design) | Higher number of childbirths may have little or no effect on lung cancer incidence |
| Melanoma incidence (1) | 3542/5657455, Up to 45 | 0.88 (0.68-1.12) | 10 | 1 fewer (3 fewer to 1 more) | LOW (due to observational design) | Higher number of childbirths may have little or no effect on melanoma incidence |
| Breast cancer incidence (1) | 14688/5657455, Up to 45 | 0.94 (0.86-1.06) | 90 | 5 fewer (13 fewer to 5 more) | LOW (due to observational design) | Higher number of childbirths may have little or no effect on breast cancer incidence |
| Cervix cancer incidence (1) | 512/NR, Up to 45 | 0.94 (0.76-1.17) | 7 | 0 fewer (2 fewer to 1 more) | LOW (due to observational design) | Higher number of childbirths may have little or no effect on cervix cancer incidence |
| Ovary cancer incidence (1) | 1983/5657455, Up to 45 | 0.97 (0.70-1.37) | 9 | 0 fewer (3 fewer to 3 more) | LOW (due to observational design) | Higher number of childbirths may have little or no effect on ovary cancer incidence |
| Prostate cancer incidence (1) | 12536/5657455, Up to 45 | 0.94 (0.86-1.03) | 89 | 5 fewer (12 fewer to 3 more) | LOW (due to observational design) | Higher number of childbirths may have little or no effect on prostate cancer incidence |
| Testis cancer incidence (2) | 674/>5,657,455, Up to 45 | 0.77 (0.57-1.04) | 4 | 1 fewer (2 fewer to 0 fewer) | LOW (due to observational design) | Higher number of childbirths may have little or no effect on testis cancer incidence |
| Kidney cancer incidence (1) | 1900/5657455, Up to 45 | 0.97 (0.68-1.37) | 8 | 0 fewer (3 fewer to 3 more) | LOW (due to observational design) | Higher number of childbirths may have little or no effect on kidney cancer incidence |
| Bladder cancer incidence (1) | 3046/5657455, Up to 45 | 0.83 (0.66-1.06) | 7 | 1 fewer (2 fewer to 0 fewer) | LOW (due to observational design) | Higher number of childbirths may have little or no effect on bladder cancer incidence |
| Thyroid cancer incidence (1) | 445/5657455, Up to 45 | 1.40 (0.70-2.80) | 6 | 2 more (2 fewer to 11 more) | LOW (due to observational design) | Higher number of childbirths may have little or no effect on thyroid cancer incidence |
| Brain and CNS cancer incidence (1) | 2756/5657455, Up to 45 | 1.19 (0.88-1.56) | 4 | 1 more (0 fewer to 2 more) | LOW (due to observational design) | Higher number of childbirths may have little or no effect on brain and CNS cancer incidence |
| Multiple myeloma incidence (1) | 867/5657455, Up to 45 | 0.88 (0.55-1.37) | 4 | 0 fewer (2 fewer to 1 more) | LOW (due to observational design) | Higher number of childbirths may have little or no effect on multiple myeloma incidence |
| Leukemia incidence (1) | 230/NR, Up to 45 | 0.98 (0.65-1.46) | 6 | 0 fewer (2 fewer to 3 more) | LOW (due to observational design) | Higher number of childbirths may have little or no effect on leukemia incidence |
| Lymphoma incidence (1) | 2640/5657455, Up to 45 | 0.91 (0.68-1.23) | 10 | 1 fewer (3 fewer to 2 more) | LOW (due to observational design) | Higher number of childbirths may have little or no effect on lymphoma incidence |
| Eye cancer incidence (1) | 234/5657455, Up to 45 | 0.88 (0.37-2.10) | 1 | 0 fewer (1 fewer to 1 more) | LOW (due to observational design) | Higher number of childbirths may have little or no effect on eye cancer incidence |
| Bone cancer incidence (1) | 84/5657455, Up to 45 | 1.40 (0.36-10.22) | 1 | 0 fewer (1 fewer to 9 more) | LOW (due to observational design) | Higher number of childbirths may have little or no effect on bone cancer incidence |
| Connective and soft tissue cancer incidence (1) | 415/5657455, Up to 45 | 0.83 (0.44-1.60) | 2 | 0 fewer (1 fewer to 1 more) | LOW (due to observational design) | Higher number of childbirths may have little or no effect on connective and soft tissue cancer incidence |
| Overall cancer mortality (1) | NR/NR, Up to 34 | 0.94 (0.75-1.17) | 96 | 6 fewer (24 fewer to 16 more) | VERY Low (due to observational design, imprecision) ^c^ | We are uncertain of the effects of higher number of childbirths on overall cancer mortality |
| Stomach cancer mortality (1) | 19/1,272, Up to 25 | 2.83 (0.64-12.43) | 5 | 9 more (2 fewer to 57 more) | VERY LOW (due to observational design, risk of bias, imprecision) ^d,e^ | We are uncertain of the effects of higher number of childbirths on stomach cancer mortality |
| **Longer breastfeeding duration compared to shorter duration** | | | | | | |
| Esophageal cancer incidence (1) | 1055/548741, Mean 12.7 | 0.88 (0.77-1.01) | 3 | 0 fewer (1 fewer to 0 fewer) | VERY LOW (due to observational design, risk of bias) ^d^ | We are uncertain of the effects of higher breastfeeding duration on esophageal cancer incidence |
| Stomach cancer incidence (1) | 848/548741, Mean 12.7 | 1.13 (0.97-1.33) | 7 | 1 more (0 fewer to 2 more) | VERY LOW (due to observational design, risk of bias) ^d^ | We are uncertain of the effects of higher breastfeeding duration on stomach cancer incidence |
| Pancreatic cancer incidence (1) | 2011/548741, Mean 12.7 | 0.96 (0.87-1.05) | 8 | 0 fewer (1 fewer to 0 fewer) | VERY LOW (due to observational design, risk of bias) ^d^ | We are uncertain of the effects of higher breastfeeding duration on pancreatic cancer incidence |
| Lung cancer incidence (1) | 6804/548741, Mean 12.7 | 1.03 (0.98-1.09) | 33 | 1 more (1 fewer to 3 more) | VERY LOW (due to observational design, risk of bias) ^d^ | We are uncertain of the effects of higher breastfeeding duration on lung cancer incidence |
| Melanoma incidence (1) | 3410/548741, Mean 12.7 | 1.00 (0.93-1.08) | 10 | 0 fewer (1 fewer to 1 more) | VERY LOW (due to observational design, risk of bias) ^d^ | We are uncertain of the effects of higher breastfeeding duration on melanoma incidence |
| Breast cancer incidence (4) | 26534/>548,741, Up to 6 | 1.06 (0.94-1.20) | 90 | 5 more (5 fewer to 18 more) | VERY LOW (due to observational design, risk of bias, imprecision) ^d,e^ | We are uncertain of the effects of higher breastfeeding duration on breast cancer incidence |
| Uterine corpus cancer incidence (2) | 5104/>548,741, Up to 28 | 1.00 (0.94-1.06) | 16 | 0 fewer (1 fewer to 1 more) | LOW (due to observational design) | Higher breastfeeding duration may have little or no effect on uterine corpus cancer incidence |
| Ovary cancer incidence (1) | 3598/548741, Mean 12.7 | 0.94 (0.88-1.02) | 9 | 1 fewer (1 fewer to 0 fewer) | VERY LOW (due to observational design, risk of bias) ^d^ | We are uncertain of the effects of higher breastfeeding duration on ovary cancer incidence |
| Lymphoma incidence (1) | 3082/548741, Mean 12.7 | 1.07 (0.98-1.16) | 10 | 1 more (0 fewer to 2 more) | VERY LOW (due to observational design, risk of bias) ^d^ | We are uncertain of the effects of higher breastfeeding duration on lymphoma cancer incidence |

RR, relative risk; CI, confidence interval; NR, not reported

^a^ Cumulative risk between 20 to 74 years the International Agency Research on Cancer online analysis system.

^b^ 3/3 studies at high risk of bias, primarily due to inappropriate measurement of reproductive factor and inadequate adjustment for confounders

^c^ Confidence interval around absolute effect includes both appreciable benefit and appreciable harm.

^d^ Study at high risk of bias for inappropriate measurement of reproductive factor and inadequate adjustment for confounders

^e^ Confidence interval around absolute effect includes both appreciable harm and no appreciable effect.

^f^ Confidence interval around absolute effect includes both appreciable benefit and no appreciable effect.
