# Supplementary material for: Impact of maternal reproductive factors on cancer risks of offspring: A systematic review and meta-analysis of cohort studies
Source: PLoS One. 2020 Mar 30;15(3):e0230721. doi: 10.1371/journal.pone.0230721 (PMC7105118; doi:10.1371/journal.pone.0230721)
Supplement: S9 Table — (DOCX) [file pone.0230721.s009.docx]

**S9 Table. Maternal reproductive factors and cancer incidence and mortality in adulthood**

| **Type of cancer** | **Risk of bias** | **No of studies** | **No of participants** | **No of cases** | **Follow-up year** | **Random effect**  **RR (95% CI)** | **I^2^, %**  **(p-value)** | **Interaction p-value** |
| --- | --- | --- | --- | --- | --- | --- | --- | --- |
| **Higher maternal age at childbirth compared to 25 to 29 maternal age** | | | | | | | | |
| Colorectum cancer incidence | Low | 1 | 16,839 | 118 | Median 31 | 0.83 (0.46-1.51) | NA | NA |
| Breast cancer incidence | High | 3 | >11,754 | 3,156 | Up to 28 | 1.03 (0.88-1.20) | 0.0 (0.754) | NA |
| Prostate cancer incidence | High | 1 | 1,089 | 70 | Up to 42 | 1.10 (0.56-2.17) | NA | NA |
| Multiple myeloma incidence | Low | 1 | NR | 37 | Mean 11 | 4.53 (1.35-15.16) | NA | NA |
| Leukemia incidence | Low | 1 | NR | 20 | Mean 11 | 1.19 (0.28-5.08) | NA | NA |
| Lymphoma incidence | Low | 1 | NR | 215 | Mean 11 | 0.87 (0.49-1.54) | NA | NA |
| Breast cancer mortality | High | 1 | 106,808 | 555 | Up to 13 | 1.38 (0.87-2.21) | NA | NA |
| **Lower maternal age at childbirth compared to 25 to 29 maternal age** | | | | | | | | |
| Colorectum cancer incidence | Low | 1 | 21,325 | 180 | Median 31 | 1.11 (0.82-1.49) | NA | NA |
| Breast cancer incidence | High | 3 | >14,498 | 3,020 | Up to 28 | 0.93 (0.84-1.02) | 0.0 (0.772) | NA |
| Prostate cancer incidence | High | 1 | 1,009 | 61 | Up to 42 | 1.00 (0.63-1.60) | NA | NA |
| Multiple myeloma incidence | Low | 1 | NR | 68 | Mean 11 | 1.32 (0.75-2.32) | NA | NA |
| Leukemia incidence | Low | 1 | NR | 47 | Mean 11 | 2.08 (1.05-4.12) | NA | NA |
| Lymphoma incidence | Low | 1 | NR | 339 | Mean 11 | 0.84 (0.65-1.08) | NA | NA |
| Breast cancer mortality | High | 1 | 128,437 | 655 | Up to 13 | 1.06 (0.87-1.30) | NA | NA |
| **Higher birth order compared to lower birth order** | | | | | | | | |
| Overall cancer incidence | Low | 1 | 11,314,910 | 154,854 | Up to 45 | 0.88 (0.80-1.00) | NA | NA |
| Esophagus cancer incidence | Low | 1 | 11,314,910 | 1,366 | Up to 45 | 0.70 (0.42-1.16) | NA | NA |
| Gastric cancer incidence | Low | 1 | 11,314,910 | 2,524 | Up to 45 | 1.23 (0.86-1.69) | NA | NA |
| Colorectum cancer incidence | Low | 2 | 11,334,451 | 8,658 | Up to 45 | 0.90 (0.74-1.08) | 0.0 (0.488) | NA |
| Liver cancer incidence | Low | 1 | 11,314,910 | 2,548 | Up to 45 | 0.78 (0.55-1.12) | NA | NA |
| Pancreatic cancer incidence | Low | 1 | 11,314,910 | 3,090 | Up to 45 | 0.80 (0.53-1.23) | NA | NA |
| Larynx cancer incidence | Low | 1 | 11,314,910 | 760 | Up to 45 | 1.16 (0.57-2.30) | NA | NA |
| Lung cancer incidence | Low | 1 | 11,314,910 | 11,088 | Up to 45 | 1.09 (0.91-1.30) | NA | NA |
| Melanoma incidence | Low | 1 | 11,314,910 | 7,084 | Up to 45 | 0.66 (0.53-0.80) | NA | NA |
| Breast cancer incidence |  | 2 | >11,314,910 | 29,844 | Up to 45 | 0.96 (0.88-1.06) | 12.6 (0.285) | NA |
|  | Low | 1 | 11,314,910 | 29,376 | Up to 45 | 0.94 (0.86-1.03) | NA | NA |
|  | High | 1 | NR | 468 | Up to 9 | 1.06 (0.87-1.30) | NA | NA |
| Cervix uteri cancer incidence | Low | 1 | 11,314,910 | 1,292 | Up to 45 | 0.57 (0.36-0.91) | NA | NA |
| Corpus uteri cancer incidence | Low | 1 | 11,314,910 | 4,490 | Up to 45 | 0.66 (0.57-0.78) | NA | NA |
| Ovary cancer incidence | Low | 1 | 11,314,910 | 3,966 | Up to 45 | 1.09 (0.86-1.44) | NA | NA |
| Prostate cancer incidence | Low | 1 | 11,314,910 | 25,072 | Up to 45 | 1.09 (1.00-1.19) | NA | NA |
| Testis cancer incidence | Low | 1 | 11,314,910 | 354 | Up to 45 | 0.47 (0.22-1.06) | NA | NA |
| Kidney cancer incidence | Low | 1 | 11,314,910 | 3,800 | Up to 45 | 0.97 (0.70-1.33) | NA | NA |
| Bladder cancer incidence | Low | 1 | 11,314,910 | 6,092 | Up to 45 | 0.83 (0.68-1.06) | NA | NA |
| Thyroid cancer incidence | Low | 1 | 11,314,910 | 890 | Up to 45 | 0.49 (0.26-0.91) | NA | NA |
| Brain and CNS cancer incidence | Low | 1 | 11,314,910 | 5,512 | Up to 45 | 0.97 (0.78-1.23) | NA | NA |
| Myeloma incidence | Low | 1 | 11,314,910 | 1,734 | Up to 45 | 1.12 (0.70-1.77) | NA | NA |
| Leukemia incidence | Low | 1 | NR | 374 | Up to 45 | 1.01 (0.64-1.59) | NA | NA |
| Lymphoma incidence | Low | 1 | NR | 329 | Up to 45 | 0.57 (0.21-1.58) | NA | NA |
| Eye cancer incidence | Low | 1 | 11,314,910 | 468 | Up to 45 | 0.73 (0.36-1.52) | NA | NA |
| Bone cancer incidence | Low | 1 | 11,314,910 | 168 | Up to 45 | 1.37 (0.33-5.74) | NA | NA |
| Connective and soft tissue cancer incidence | Low | 1 | 11,314,910 | 830 | Up to 45 | 1.00 (0.55-1.77) | NA | NA |
| **Higher number of childbirths compared to smaller number of childbirths** | | | | | | | | |
| Overall cancer incidence | Low | 1 | 5,657,455 | 77,427 | Up to 45 | 0.97 (0.83-1.12) | NA | NA |
| Esophagus cancer incidence | Low | 1 | 5,657,455 | 683 | Up to 45 | 0.97 (0.55-1.64) | NA | NA |
| Gastric cancer incidence | Low | 1 | 5,657,455 | 1,262 | Up to 45 | 1.60 (1.06-2.41) | NA | NA |
| Colorectal cancer incidence | Low | 1 | 5,657,455 | 4,255 | Up to 45 | 0.86 (0.70-1.06) | NA | NA |
| Hepatic cancer incidence | Low | 1 | 5,657,455 | 1,274 | Up to 45 | 0.86 (0.57-1.26) | NA | NA |
| Pancreatic cancer incidence | Low | 1 | 5,657,455 | 1,545 | Up to 45 | 0.80 (0.53-1.19) | NA | NA |
| Larynx cancer incidence | Low | 1 | 5,657,455 | 380 | Up to 45 | 1.52 (0.75-3.05) | NA | NA |
| Lung cancer incidence | Low | 1 | 5,657,455 | 5,544 | Up to 45 | 0.86 (0.70-1.06) | NA | NA |
| Melanoma incidence | Low | 1 | 5,657,455 | 3,542 | Up to 45 | 0.88 (0.68-1.12) | NA | NA |
| Breast cancer incidence | Low | 1 | 5,657,455 | 14,688 | Up to 45 | 0.94 (0.86-1.06) | NA | NA |
| Cervix cancer incidence | Low | 1 | NR | 512 | Up to 45 | 0.94 (0.76-1.17) | NA | NA |
| Corpus uteri cancer incidence | Low | 1 | 5,657,455 | 2,966 | Up to 45 | 0.47 (0.37-0.59) | NA | NA |
| Ovary cancer incidence | Low | 1 | 5,657,455 | 1,983 | Up to 45 | 0.97 (0.70-1.37) | NA | NA |
| Prostate cancer incidence | Low | 1 | 5,657,455 | 12,536 | Up to 45 | 0.94 (0.86-1.03) | NA | NA |
| Testis cancer incidence | Low | 2 | >5,657,455 | 674 | Up to 45 | 0.77 (0.57-1.04) | 47.2 (0.108) | NA |
| Kidney cancer incidence | Low | 1 | 5,657,455 | 1,900 | Up to 45 | 0.97 (0.68-1.37) | NA | NA |
| Bladder cancer incidence | Low | 1 | 5,657,455 | 3,046 | Up to 45 | 0.83 (0.66-1.06) | NA | NA |
| Thyroid cancer incidence | Low | 1 | 5,657,455 | 445 | Up to 45 | 1.40 (0.70-2.80) | NA | NA |
| Brain and CNS cancer incidence | Low | 1 | 5,657,455 | 2,756 | Up to 45 | 1.19 (0.88-1.56) | NA | NA |
| Multiple myeloma incidence | Low | 1 | 5,657,455 | 867 | Up to 45 | 0.88 (0.55-1.37) | NA | NA |
| Leukemia incidence | Low | 1 | NR | 230 | Up to 45 | 0.98 (0.65-1.46) | NA | NA |
| Lymphoma incidence | Low | 1 | 5,657,455 | 2,640 | Up to 45 | 0.91 (0.68-1.23) | NA | NA |
| Retinoblastoma incidence | Low | 1 | 5,657,455 | 234 | Up to 45 | 0.88 (0.37-2.10) | NA | NA |
| Bone cancer incidence | Low | 1 | 5,657,455 | 84 | Up to 45 | 1.40 (0.36-10.22) | NA | NA |
| Connective and soft tissue cancer incidence | Low | 1 | 5,657,455 | 415 | Up to 45 | 0.83 (0.44-1.60) | NA | NA |
| Overall cancer mortality | Low | 1 | NR | NR | Up to 34 | 0.94 (0.75-1.17) | NA | NA |
| Stomach cancer mortality | High | 1 | 1,272 | 19 | Up to 25 | 2.83 (0.64-12.43) | NA | NA |
| Lung cancer mortality | High | 1 | 1,272 | 67 | Up to 25 | 2.18 (1.05-4.52) | NA | NA |
| **Longer breastfeeding duration compared to shorter duration** | | | | | | | | |
| Esophageal cancer incidence | High | 1 | 548,741 | 1,055 | Mean 12.7 | 0.88 (0.77-1.01) | NA | NA |
| Stomach cancer incidence | High | 1 | 548,741 | 848 | Mean 12.7 | 1.13 (0.97-1.33) | NA | NA |
| Colorectal cancer incidence | High | 1 | 548,741 | 8,651 | Mean 12.7 | 1.18 (1.12-1.24) | NA | NA |
| Pancreatic cancer incidence | High | 1 | 548,741 | 2,011 | Mean 12.7 | 0.96 (0.87-1.05) | NA | NA |
| Lung cancer incidence | High | 1 | 548,741 | 6,804 | Mean 12.7 | 1.03 (0.98-1.09) | NA | NA |
| Melanoma incidence | High | 1 | 548,741 | 3,410 | Mean 12.7 | 1.00 (0.93-1.08) | NA | NA |
| Breast cancer incidence | High | 4 | >548,741 | 26,534 | Up to 6 | 1.06 (0.94-1.20) | 34.2 (0.229) | NA |
| Uterine corpus cancer incidence |  | 2 | >548,741 | 5,104 | Up to 28 | 1.00 (0.94-1.06) | 0.0 (0.941) | NA |
|  | Low | 1 | NR | 327 | Up to 28 | 0.99 (0.77-1.29) | NA | NA |
|  | High | 1 | 548,741 | 4,777 | Mean 12.7 | 1.00 (0.94-1.07) | NA | NA |
| Ovary cancer incidence | High | 1 | 548,741 | 3,598 | Mean 12.7 | 0.94 (0.88-1.02) | NA | NA |
| Lymphoma incidence | High | 1 | 548,741 | 3,082 | Mean 12.7 | 1.07 (0.98-1.16) | NA | NA |

CI, confidence interval; NA, not applicable; NR, not reported; RR, relative risk
