# Supplementary material for: Impact of maternal reproductive factors on cancer risks of offspring: A systematic review and meta-analysis of cohort studies
Source: PLoS One. 2020 Mar 30;15(3):e0230721. doi: 10.1371/journal.pone.0230721 (PMC7105118; doi:10.1371/journal.pone.0230721)
Supplement: S10 Table — (DOCX) [file pone.0230721.s010.docx]

**S10 Table. Summary of finding for maternal reproductive factors and lifetime cancer incidence and mortality**

| **Outcomes (no of studies)** | **No of cases/participants, follow-up years** | **Relative risk (95% CI)** | **Population risk**  **(per 1000) ^a^** | **Risk difference**  **(per 1000)** | **Certainty of the evidence** | **Plain language summary** |
| --- | --- | --- | --- | --- | --- | --- |
| **Higher maternal age at childbirth compared to 25 to 29 maternal age** | | | | | | |
| Overall cancer incidence (1) | 740/159721, Mean 21.3 | 1.02 (0.79-1.31) | 202 | 4 more (42 fewer to 63 more) | VERY LOW (due to observational design, imprecision) ^b^ | We are uncertain of the effects of higher maternal age at birth on overall cancer incidence |
| Colorectal cancer incidence (1) | NR/NR, Mean 22 | 0.83 (0.58-1.18) | 13 | 2 fewer (5 fewer to 2 more) | LOW (due to observational design) | Higher maternal age at birth may have little or no effect on colorectum cancer incidence |
| Lung cancer incidence (1) | NR/NR, Mean 22 | 1.12 (0.74-1.70) | 28 | 3 more (7 fewer to 20 more) | VERY LOW (due to observational design, imprecision) ^b^ | We are uncertain of the effects of higher maternal age at birth on lung cancer incidence |
| Melanoma incidence (3) | >1,181/>2,434,912, Up to 37 | 1.20 (0.95-1.51) | 4 | 1 more (0 fewer to 2 more) | LOW (due to observational design) | Higher maternal age at birth may have little or no effect on melanoma incidence |
| Breast cancer incidence (2) | >45 />159,721, Up to 22 | 1.06 (0.90-1.26) | 50 | 3 more (5 fewer to 13 more) | LOW (due to observational design) | Higher maternal age at birth may have little or no effect on breast cancer incidence |
| Uterine corpus cancer incidence (1) | 66/NR, Up to 22 | 1.03 (0.52-2.07) | 10 | 0 fewer (5 fewer to 11 more) | LOW (due to observational design) | Higher maternal age at birth may have little or no effect on uterine corpus cancer incidence |
| Prostate cancer incidence (1) | 16/, Mean 22 | 0.98 (0.25-3.82) | 37 | 1 fewer (28 fewer to 104 more) | VERY LOW (due to observational design, imprecision) ^b^ | We are uncertain of the effects of higher maternal age at birth on prostate cancer incidence |
| Testis cancer incidence (4) | >152/NR, Up to 32 | 1.03 (0.89-1.18) | 1 | 0 fewer (0 fewer to 0 fewer) | LOW (due to observational design) | Higher maternal age at birth may have little or no effect on testis cancer incidence |
| Kidney cancer incidence (2) | >27 />159,721, Up to 22 | 1.11 (0.69-1.78) | 5 | 1 more (2 fewer to 4 more) | LOW (due to observational design) | Higher maternal age at birth may have little or no effect on kidney cancer incidence |
| Thyroid cancer incidence (1) | NR/NR, Mean 22 | 0.79 (0.53-1.17) | 7 | 1 fewer (3 fewer to 1 more) | LOW (due to observational design) | Higher maternal age at birth may have little or no effect on thyroid cancer incidence |
| Brain and CNS cancer incidence (2) | >1,349 />1,769,433, Up to 38 | 0.93 (0.84-1.04) | 4 | 0 fewer (1 fewer to 0 fewer) | LOW (due to observational design) | Higher maternal age at birth may have little or no effect on brain and CNS cancer incidence |
| Leukemia incidence (2) | 70/159721, Up to 22 | 1.34 (1.00-1.79) | 5 | 2 more (0 fewer to 4 more) | LOW (due to observational design) | Higher maternal age at birth may have little or no effect on leukemia incidence |
| Lymphoma incidence (3) | >490 />1,339,902 , Up to 22 | 1.14 (0.91-1.42) | 6 | 1 more (1 fewer to 3 more) | LOW (due to observational design) | Higher maternal age at birth may have little or no effect on lymphoma incidence |
| **Lower maternal age at childbirth compared to 25 to 29 maternal age** | | | | | | |
| Overall cancer incidence (1) | 1005/223133, Mean 21.3 | 0.89 (0.78-1.02) | 202 | 22 fewer (44 fewer to 4 more) | VERY LOW (due to observational design, imprecision) ^c^ | We are uncertain of the effects of lower maternal age at birth on overall cancer incidence |
| Colorectal cancer incidence (1) | >85 /NR, Mean 22 | 1.20 (0.85-1.71) | 13 | 3 more (2 fewer to 9 more) | LOW (due to observational design) | Lower maternal age at birth may have little or no effect on colorectal cancer incidence |
| Lung cancer incidence (1) | >86 /NR, Mean 22 | 0.89 (0.59-1.36) | 28 | 3 fewer (11 fewer to 10 more) | LOW (due to observational design) | Lower maternal age at birth may have little or no effect on lung cancer incidence |
| Melanoma incidence (3) | >1,659/>2,453,104, Up to 37 | 0.91 (0.72-1.15) | 4 | 0 fewer (1 fewer to 1 more) | LOW (due to observational design) | Lower maternal age at birth may have little or no effect on melanoma incidence |
| Breast cancer incidence (2) | >581/>223,133, Up to 22 | 0.74 (0.41-1.35) | 50 | 13 fewer (30 fewer to 18 more) | VERY LOW (due to observational design, imprecision) ^b^ | We are uncertain of the effects of lower maternal age at birth on breast cancer incidence |
| Corpus uteri cancer incidence (1) | >33/NR, Up to 22 | 0.97 (0.48-1.95) | 10 | 0 fewer (5 fewer to 10 more) | LOW (due to observational design) | Lower maternal age at birth may have little or no effect on corpus uteri cancer incidence |
| Prostate cancer incidence (1) | >8/NR, Up to 22 | 1.02 (0.26-3.98) | 37 | 1 more (27 fewer to 110 more) | VERY LOW (due to observational design, imprecision) ^b^ | We are uncertain of the effects of lower maternal age at birth on prostate cancer incidence |
| Testis cancer incidence (4) | >376/NR, Up to 31.6 | 1.00 (0.88-1.13) | 1 | 0 fewer (0 fewer to 0 fewer) | LOW (due to observational design) | Lower maternal age at birth may have little or no effect on testis cancer incidence |
| Kidney cancer incidence (2) | >86/>223,133, Up to 22 | 0.94 (0.62-1.44) | 5 | 0 fewer (2 fewer to 2 more) | LOW (due to observational design) | Lower maternal age at birth may have little or no effect on kidney cancer incidence |
| Thyroid cancer incidence (1) | >65/NR, Mean 22 | 1.27 (0.85-1.87) | 7 | 2 more (1 fewer to 6 more) | LOW (due to observational design) | Lower maternal age at birth may have little or no effect on thyroid cancer incidence |
| Brain and CNS cancer incidence (2) | >1,952/>2,012,268, Up to 38 | 1.30 (0.94-1.13) | 4 | 1 more (0 fewer to 1 more) | LOW (due to observational design) | Lower maternal age at birth may have little or no effect on brain and CNS cancer incidence |
| Leukemia incidence (2) | >214/>223,133, Up to 22 | 0.86 (0.63-1.18) | 5 | 1 fewer (2 fewer to 1 more) | LOW (due to observational design) | Lower maternal age at birth may have little or no effect on leukemia incidence |
| Lymphoma incidence (3) | >726/1337332, Up to 22 | 0.83 (0.69-1.01) | 6 | 1 fewer (2 fewer to 0 fewer) | LOW (due to observational design) | Lower maternal age at birth may have little or no effect on lymphoma incidence |
| **Higher birth order compared to lower birth order** | | | | | | |
| Overall cancer incidence (1) | 269792/11314910, Up to 45 | 0.91 (0.86-1.00) | 202 | 18 fewer (28 fewer to 0 fewer) | LOW (due to observational design, imprecision) ^d^ | We are uncertain of the effects of higher birth order on overall cancer incidence |
| Esophagus cancer incidence (1) | 1580/11314910, Up to 45 | 0.70 (0.44-1.16) | 8 | 2 fewer (4 fewer to 1 more) | LOW (due to observational design) | Higher birth order may have little or no effect on esophagus cancer incidence |
| Gastric cancer incidence (1) | 3510/11314910, Up to 45 | 1.19 (0.83-1.73) | 13 | 2 more (2 fewer to 9 more) | LOW (due to observational design) | Higher birth order may have little or no effect on gastric cancer incidence |
| Colorectal cancer incidence (1) | 12242/11314910, Up to 45 | 0.91 (0.75-1.06) | 13 | 1 fewer (3 fewer to 1 more) | LOW (due to observational design) | Higher birth order may have little or no effect on colorectal cancer incidence |
| Liver cancer incidence (1) | 3386/11314910, Up to 45 | 0.83 (0.61-1.12) | 11 | 2 fewer (4 fewer to 1 more) | LOW (due to observational design) | Higher birth order may have little or no effect on liver cancer incidence |
| Pancreatic cancer incidence (1) | 3614/11314910, Up to 45 | 0.80 (0.59-1.09) | 6 | 1 fewer (2 fewer to 1 more) | LOW (due to observational design) | Higher birth order may have little or no effect on pancreatic cancer incidence |
| Larynx cancer incidence (1) | 1018/11314910, Up to 45 | 1.00 (0.57-1.69) | 3 | 0 fewer (1 fewer to 2 more) | LOW (due to observational design) | Higher birth order may have little or no effect on larynx cancer incidence |
| Melanoma incidence (2) | >914/>1,228,889, Up to 46 | 0.51 (0.21-1.24) | 4 | 2 fewer (3 fewer to 1 more) | LOW (due to observational design) | Higher birth order may have little or no effect on melanoma incidence |
| Breast cancer incidence (1) | 49038/11314910, Up to 45 | 1.00 (0.75-1.37) | 50 | 0 fewer (13 fewer to 19 more) | LOW (due to observational design, imprecision) ^b^ | We are uncertain of the effects of higher birth order on breast cancer incidence |
| Cervix uteri cancer incidence (1) | 7704/11314910, Up to 45 | 0.80 (0.61-1.06) | 14 | 3 fewer (5 fewer to 1 more) | LOW (due to observational design) | Higher birth order may have little or no effect on uterine cervix cancer incidence |
| Ovary cancer incidence (1) | 7506/11314910, Up to 45 | 1.03 (0.83-1.30) | 7 | 0 fewer (1 fewer to 2 more) | LOW (due to observational design) | Higher birth order may have little or no effect on ovary cancer incidence |
| Kidney cancer incidence (1) | 6342/11314910, Up to 45 | 0.97 (0.78-1.19) | 5 | 0 fewer (1 fewer to 1 more) | LOW (due to observational design) | Higher birth order may have little or no effect on kidney cancer incidence |
| Bladder cancer incidence (1) | 8042/11314910, Up to 45 | 0.91 (0.75-1.12) | 7 | 1 fewer (2 fewer to 1 more) | LOW (due to observational design) | Higher birth order may have little or no effect on urinary cancer incidence |
| Brain and CNS cancer incidence (1) | 19400/11314910, Up to 45 | 1.03 (0.88-1.19) | 4 | 0 fewer (0 fewer to 1 more) | LOW (due to observational design) | Higher birth order may have little or no effect on brain and CNS cancer incidence |
| Multiple myeloma incidence (1) | 893/NR, Up to 45 | 0.77 (0.46-1.30) | 2 | 0 fewer (1 fewer to 1 more) | LOW (due to observational design) | Higher birth order may have little or no effect on multiple myeloma incidence |
| Leukemia incidence (1) | 4002/NR, Up to 45 | 0.81 (0.62-1.07) | 5 | 1 fewer (2 fewer to 0 fewer) | LOW (due to observational design) | Higher birth order may have little or no effect on leukemia incidence |
| Lymphoma incidence (2) | 3985/NR, Up to 45 | 0.63 (0.20-2.00) | 6 | 2 fewer (5 fewer to 6 more) | VERY LOW (due to observational design, inconsistency) ^e^ | We are uncertain of the effects of higher birth order on lymphoma incidence |
| Eye cancer incidence (1) | 1548/11314910, Up to 45 | 1.03 (0.68-1.52) | 1 | 0 fewer (0 fewer to 1 more) | LOW (due to observational design) | Higher birth order may have little or no effect on eye cancer incidence |
| Bone cancer incidence (1) | 1992/11314910, Up to 45 | 1.26 (0.86-1.82) | 1 | 0 fewer (0 fewer to 1 more) | LOW (due to observational design) | Higher birth order may have little or no effect on bone cancer incidence |
| **Higher number of childbirths compared to smaller number of childbirths** | | | | | | |
| Overall cancer incidence (1) | 134896/5657455, Up to 45 | 0.97 (0.88-1.06) | 202 | 6 fewer (24 fewer to 12 more) | LOW (due to observational design, imprecision) ^d^ | We are uncertain of the effects of higher number of childbirths on overall cancer incidence |
| Esophagus cancer incidence (1) | 790/5657455, Up to 45 | 0.78 (0.46-1.33) | 8 | 2 fewer (4 fewer to 3 more) | LOW (due to observational design) | Higher number of childbirths may have little or no effect on esophagus cancer incidence |
| Colorectal cancer incidence (1) | 2921/NR, Up to 46 | 0.98 (0.88-1.10) | 13 | 0 fewer (2 fewer to 1 more) | LOW (due to observational design) | Higher number of childbirths may have little or no effect on colorectal cancer incidence |
| Liver cancer incidence (1) | 843/NR, Up to 46 | 1.01 (0.88-1.17) | 11 | 0 fewer (1 fewer to 2 more) | LOW (due to observational design) | Higher number of childbirths may have little or no effect on liver cancer incidence |
| Pancreas cancer incidence (1) | 918/NR, Up to 46 | 1.11 (0.98-1.26) | 6 | 1 more (0 fewer to 2 more) | LOW (due to observational design) | Higher number of childbirths may have little or no effect on pancreatic cancer incidence |
| Larynx cancer incidence (1) | 509/5657455, Up to 45 | 1.52 (0.73-3.18) | 3 | 2 more (1 fewer to 7 more) | LOW (due to observational design) | Higher number of childbirths may have little or no effect on larynx cancer incidence |
| Breast cancer incidence (1) | 10959/NR, Up to 46 | 0.96 (0.93-1.00) | 50 | 2 fewer (4 fewer to 0 fewer) | LOW (due to observational design) | Higher number of childbirths may have little or no effect on breast cancer incidence |
| Ovary cancer incidence (1) | 1765/NR, Up to 46 | 0.98 (0.90-1.06) | 7 | 0 fewer (1 fewer to 0 fewer) | LOW (due to observational design) | Higher number of childbirths may have little or no effect on ovary cancer incidence |
| Prostate cancer incidence (1) | 5910/NR, Up to 46 | 0.97 (0.91-1.03) | 37 | 1 fewer (3 fewer to 1 more) | LOW (due to observational design) | Higher number of childbirths may have little or no effect on prostate cancer incidence |
| Kidney cancer incidence (1) | 1380/NR, Up to 46 | 1.00 (0.86-1.16) | 5 | 0 fewer (1 fewer to 1 more) | LOW (due to observational design) | Higher number of childbirths may have little or no effect on kidney cancer incidence |
| Urinary bladder cancer incidence (1) | 1990/NR, Up to 46 | 1.01 (0.89-1.15) | 7 | 0 fewer (1 fewer to 1 more) | LOW (due to observational design) | Higher number of childbirths may have little or no effect on urinary cancer incidence |
| Thyroid cancer incidence (1) | 787/NR, Up to 46 | 1.04 (0.88-1.22) | 7 | 0 fewer (1 fewer to 2 more) | LOW (due to observational design) | Higher number of childbirths may have little or no effect on thyroid cancer incidence |
| CNS cancer incidence (1) | 3466/NR, Up to 46 | 1.06 (0.99-1.13) | 4 | 0 fewer (0 fewer to 1 more) | LOW (due to observational design) | Higher number of childbirths may have little or no effect on CNS cancer incidence |
| Leukemia incidence (1) | 2884/NR, Up to 45 | 1.13 (1.00-1.27) | 5 | 1 more (0 fewer to 1 more) | LOW (due to observational design) | Higher number of childbirths may have little or no effect on leukemia incidence |
| Lymphoma incidence (2) | 3094/NR, Up to 45 | 1.05 (0.92-1.20) | 6 | 0 fewer (0 fewer to 1 more) | LOW (due to observational design) | Higher number of childbirths may have little or no effect on lymphoma incidence |
| Eye cancer incidence (1) | 774/5657455, Up to 45 | 0.91 (0.51-1.64) | 1 | 0 fewer (0 fewer to 1 more) | LOW (due to observational design) | Higher number of childbirths may have little or no effect on eye cancer incidence |
| Bone cancer incidence (1) | 259/NR, Up to 46 | 0.85 (0.63-1.13) | 1 | 0 fewer (0 fewer to 0 fewer) | LOW (due to observational design) | Higher number of childbirths may have little or no effect on bone cancer incidence |
| Connective and soft tissue cancer incidence (1) | 565/NR, Up to 46 | 0.90 (0.73-1.12) | 2 | 0 fewer (1 fewer to 0 fewer) | LOW (due to observational design) | Higher number of childbirths may have little or no effect on connective and soft tissue cancer incidence |
| **Longer breastfeeding duration compared to shorter duration** | | | | | | |
| Overall cancer incidence (1) | NR/3844, Mean 48.3 | 1.07 (0.89-1.28) | 202 | 14 more (22 fewer to 57 more) | VERY LOW (due to observational design, imprecision) ^b^ | We are uncertain of the effects of higher breastfeeding duration on overall cancer incidence |
| Gastric cancer incidence (1) | NR/3844, Mean 48.3 | 1.22 (0.47-3.15) | 13 | 3 more (7 fewer to 28 more) | VERY LOW (due to observational design, imprecision) ^d^ | We are uncertain of the effects of higher breastfeeding duration on gastric cancer incidence |
| Colorectal cancer incidence (1) | NR/3844, Mean 48.3 | 0.86 (0.45-1.63) | 13 | 2 fewer (7 fewer to 8 more) | LOW (due to observational design) | Higher breastfeeding duration may have little or no effect on colorectal cancer incidence |
| Breast cancer incidence (1) | NR/NR, Mean 48.3 | 1.62 (0.89-2.94) | 50 | 31 more (6 fewer to 97 more) | VERY LOW (due to observational design, imprecision) ^d^ | We are uncertain of the effects of higher breastfeeding duration on breast cancer incidence |
| Prostate cancer incidence (1) | NR/NR, Mean 48.3 | 1.43 (0.58-3.52) | 37 | 16 more (16 fewer to 93 more) | VERY LOW (due to observational design, imprecision) ^b^ | We are uncertain of the effects of higher breastfeeding duration on prostate cancer incidence |
| Overall cancer mortality (1) | NR/3844, Mean 48.3 | 1.09 (0.86-1.37) | 106 | 10 more (15 fewer to 39 more) | VERY LOW (due to observational design, imprecision) ^d^ | We are uncertain of the effects of higher breastfeeding duration on overall cancer mortality |
| Gastric cancer mortality (1) | NR/3844, Mean 48.3 | 1.43 (0.51-4.01) | 10 | 4 more (5 fewer to 30 more) | VERY LOW (due to observational design, imprecision) ^d^ | We are uncertain of the effects of higher breastfeeding duration on gastric cancer mortality |
| Colorectal cancer mortality (1) | NR/3844, Mean 48.3 | 0.96 (0.41-2.21) | 5 | 0 fewer (3 fewer to 6 more) | LOW (due to observational design) | Higher breastfeeding duration may have little or no effect on colorectal cancer mortality |
| Breast cancer mortality (1) | NR/NR, Mean 48.3 | 1.53 (0.61-3.83) | 14 | 7 more (5 fewer to 40 more) | VERY LOW (due to observational design, imprecision) ^d^ | We are uncertain of the effects of higher breastfeeding duration on breast cancer mortality |
| Prostate cancer mortality (1) | NR/NR, Mean 48.3 | 1.34 (0.42-4.22) | 6 | 2 more (3 fewer to 19 more) | VERY LOW (due to observational design, imprecision) ^d^ | We are uncertain of the effects of higher breastfeeding duration on prostate cancer mortality |

^a^ Lifetime cumulative risk from Globocan 2018 statistics (Farley et al., 2019).

^b^ Confidence interval around absolute effect includes both appreciable benefit and appreciable harm.

^c^ Confidence interval around absolute effect includes both appreciable benefit and no appreciable effect.

^d^ Confidence interval around absolute effect includes both appreciable harm and no appreciable effect.

^e^ I^2^=75.9%, Q-test p-value=0.042
