# Supplementary material for: Impact of maternal reproductive factors on cancer risks of offspring: A systematic review and meta-analysis of cohort studies
Source: PLoS One. 2020 Mar 30;15(3):e0230721. doi: 10.1371/journal.pone.0230721 (PMC7105118; doi:10.1371/journal.pone.0230721)
Supplement: S11 Table — (DOCX) [file pone.0230721.s011.docx]

**S11 Table. Maternal reproductive factors and cancer incidence and mortality in lifetime**

| **Type of cancer** | **Risk of bias** | **No of studies** | **No of participants** | **No of cases** | **Follow-up year** | **Random effect**  **RR (95% CI)** | **I^2^, %**  **(p-value)** | **Interaction p-value** |
| --- | --- | --- | --- | --- | --- | --- | --- | --- |
| **Higher maternal age at childbirth compared to 25 to 29 maternal age** | | | | | | | | |
| Overall cancer incidence | Low | 1 | 159,721 | 740 | Mean 21.3 | 1.02 (0.79-1.31) | NA | NA |
| Colorectal cancer incidence | Low | 1 | NR | NR | Mean 22 | 0.83 (0.58-1.18) | NA | NA |
| Lung cancer incidence | Low | 1 | NR | NR | Mean 22 | 1.12 (0.74-1.70) | NA | NA |
| Melanoma incidence | Low | 3 | >2,434,912 | >1,181 | Up to 37 | 1.20 (0.95-1.51) | 19.1 (0.252) | NA |
| Breast cancer incidence | Low | 2 | >159,721 | >45 | Up to 22 | 1.06 (0.90-1.26) | 0.0 (0.663) | NA |
| Uterine cervix cancer incidence | Low | 2 | >159,721 | >25 | Up to 22 | 0.66 (0.51-0.85) | 0.0 (0.889) | NA |
| Uterine corpus cancer incidence | Low | 1 | NR | 66 | Up to 22 | 1.03 (0.52-2.07) | NA | NA |
| Prostate cancer incidence | Low | 1 |  | 16 | Mean 22 | 0.98 (0.25-3.82) | NA | NA |
| Testis cancer incidence |  | 4 | NR | >152 | Up to 32 | 1.03 (0.89-1.18) | 0.0 (0.701) | 0.541 |
|  | Low | 3 | NR | >152 | Up to 32 | 0.97 (0.76-1.23) | 0.0 (0.593) | NA |
|  | High | 1 | NR | NR | Mean 15.6 | 1.06 (0.89-1.26) | NA | NA |
| Kidney cancer incidence | Low | 2 | >159,721 | >27 | Up to 22 | 1.11 (0.69-1.78) | 0.0 (0.446) | NA |
| Thyroid cancer incidence | Low | 1 | NR | NR | Mean 22 | 0.79 (0.53-1.17) | NA | NA |
| Brain and CNS cancer incidence | Low | 2 | >1,769,433 | >1,349 | Up to 38 | 0.93 (0.84-1.04) | 0.0 (0.546) | NA |
| Leukemia incidence | Low | 2 | 159,721 | 70 | Up to 22 | 1.34 (1.00-1.79) | 0.0 (0.826) | NA |
| Lymphoma incidence | Low | 3 | >1,339,902 | >490 | Up to 22 | 1.14 (0.91-1.42) | 0.0 (0.721) | NA |
| **Lower maternal age at childbirth compared to 25 to 29 maternal age** | | | | | | | | |
| Overall cancer incidence | Low | 1 | 223,133 | 1,005 | Mean 21.3 | 0.89 (0.78-1.02) | NA | NA |
| Colorectal cancer incidence | Low | 1 | NR | >85 | Mean 22 | 1.20 (0.85-1.71) | NA | NA |
| Lung cancer incidence | Low | 1 | NR | >86 | Mean 22 | 0.89 (0.59-1.36) | NA | NA |
| Melanoma incidence | Low | 3 | >2,453,104 | >1,659 | Up to 37 | 0.91 (0.72-1.15) | 35.8 (0.261) | NA |
| Breast cancer incidence | Low | 2 | >223,133 | >581 | Up to 22 | 0.74 (0.41-1.35) | 67.3 (0.081) | NA |
| Cervix cancer incidence | Low | 2 | >223,133 | >203 | Up to 22 | 1.61 (1.25-2.07) | 0.0 (0.320) | NA |
| Corpus uteri cancer incidence | Low | 1 | NR | >33 | Up to 22 | 0.97 (0.48-1.95) | NA | NA |
| Prostate cancer incidence | Low | 1 | NR | >8 | Up to 22 | 1.02 (0.26-3.98) | NA | NA |
| Testis cancer incidence |  | 4 | NR | >376 | Up to 31.6 | 1.00 (0.88-1.13) | 0.0 (0.717) | 0.321 |
|  | Low | 3 | NR | >376 | Up to 31.6 | 1.07 (0.88-1.30) | 0.0 (0.831) | NA |
|  | High | 1 | NR | NR | Mean 15.6 | 0.94 (0.79-1.11) | NA | NA |
| Kidney cancer incidence | Low | 2 | >223,133 | >86 | Up to 22 | 0.94 (0.62-1.44) | 0.0 (0.854) | NA |
| Thyroid cancer incidence | Low | 1 | NR | >65 | Mean 22 | 1.27 (0.85-1.87) | NA | NA |
| Brain and CNS cancer incidence | Low | 2 | >2,012,268 | >1,952 | Up to 38 | 1.30 (0.94-1.13) | 1.55 (0.314) | NA |
| Leukemia incidence | Low | 2 | >223,133 | >214 | Up to 22 | 0.86 (0.63-1.18) | 27.78 (0.239) | NA |
| Lymphoma incidence | Low | 3 | 1,337,332 | >726 | Up to 22 | 0.83 (0.69-1.01) | 0.0 (0.976) | NA |
| **Higher birth order compared to lower birth order** | | | | | | | | |
| Overall cancer incidence | Low | 1 | 11,314,910 | 269,792 | Up to 45 | 0.91 (0.86-1.00) | NA | NA |
| Esophagus cancer incidence | Low | 1 | 11,314,910 | 1,580 | Up to 45 | 0.70 (0.44-1.16) | NA | NA |
| Gastric cancer incidence | Low | 1 | 11,314,910 | 3,510 | Up to 45 | 1.19 (0.83-1.73) | NA | NA |
| Colorectal cancer incidence | Low | 1 | 11,314,910 | 12,242 | Up to 45 | 0.91 (0.75-1.06) | NA | NA |
| Liver cancer incidence | Low | 1 | 11,314,910 | 3,386 | Up to 45 | 0.83 (0.61-1.12) | NA | NA |
| Pancreatic cancer incidence | Low | 1 | 11,314,910 | 3,614 | Up to 45 | 0.80 (0.59-1.09) | NA | NA |
| Larynx cancer incidence | Low | 1 | 11,314,910 | 1,018 | Up to 45 | 1.00 (0.57-1.69) | NA | NA |
| Lung cancer incidence | Low | 1 | 11,314,910 | 13,174 | Up to 45 | 1.26 (1.06-1.44) | NA | NA |
| Melanoma incidence | Low | 2 | >1,228,889 | >914 | Up to 46 | 0.51 (0.21-1.24) | 49.9 (0.158) | NA |
| Breast cancer incidence | Low | 1 | 11,314,910 | 49,038 | Up to 45 | 1.00 (0.75-1.37) | NA | NA |
| Cervix uteri cancer incidence | Low | 1 | 11,314,910 | 7,704 | Up to 45 | 0.80 (0.61-1.06) | NA | NA |
| Corpus uteri cancer incidence | Low | 1 | NR | NR | Up to 46 | 0.60 (0.46-0.78) | NA | NA |
| Ovary cancer incidence | Low | 1 | 11,314,910 | 7,506 | Up to 45 | 1.03 (0.83-1.30) | NA | NA |
| Prostate cancer incidence | Low | 1 | NR | NR | Up to 46 | 1.38 (1.23-1.55) | NA | NA |
| Testis cancer incidence |  | 2 | >248,828 | NR | Up to 46 | 0.72 (0.58-0.89) | 0.0 (0.554) | NA |
|  | Low | 1 | NR | NR | Up to 46 | 0.70 (0.56-0.89) | NA | NA |
|  | High | 1 | 248,828 | NR | Up to 6 | 0.83 (0.50-1.40) | NA | NA |
| Kidney cancer incidence | Low | 1 | 11,314,910 | 6,342 | Up to 45 | 0.97 (0.78-1.19) | NA | NA |
| Bladder cancer incidence | Low | 1 | 11,314,910 | 8,042 | Up to 45 | 0.91 (0.75-1.12) | NA | NA |
| Thyroid cancer incidence | Low | 1 | 11,314,910 | 4,320 | Up to 45 | 0.61 (0.47-0.78) | NA | NA |
| Brain and CNS cancer incidence | Low | 1 | 11,314,910 | 19,400 | Up to 45 | 1.03 (0.88-1.19) | NA | NA |
| Multiple myeloma incidence | Low | 1 | NR | 893 | Up to 45 | 0.77 (0.46-1.30) | NA | NA |
| Leukemia incidence | Low | 1 | NR | 4,002 | Up to 45 | 0.81 (0.62-1.07) | NA | NA |
| Lymphoma incidence | Low | 2 | NR | 3985 | Up to 45 | 0.63 (0.20-2.00) | 75.9 (0.042) | NA |
| Eye cancer incidence | Low | 1 | 11,314,910 | 1,548 | Up to 45 | 1.03 (0.68-1.52) | NA | NA |
| Bone cancer incidence | Low | 1 | 11,314,910 | 1,992 | Up to 45 | 1.26 (0.86-1.82) | NA | NA |
| Connective and soft tissue cancer incidence | Low | 1 | 11,314,910 | 3,126 | Up to 45 | 0.73 (0.55-0.97) | NA | NA |
| **Higher number of childbirths compared to smaller number of childbirths** | | | | | | | | |
| Overall cancer incidence | Low | 1 | 5,657,455 | 134,896 | Up to 45 | 0.97 (0.88-1.06) | NA | NA |
| Esophagus cancer incidence | Low | 1 | 5,657,455 | 790 | Up to 45 | 0.78 (0.46-1.33) | NA | NA |
| Stomach cancer incidence | Low | 1 | NR | 946 | Up to 46 | 1.48 (1.31-1.67) | NA | NA |
| Colorectal cancer incidence | Low | 1 | NR | 2,921 | Up to 46 | 0.98 (0.88-1.10) | NA | NA |
| Liver cancer incidence | Low | 1 | NR | 843 | Up to 46 | 1.01 (0.88-1.17) | NA | NA |
| Pancreas cancer incidence | Low | 1 | NR | 918 | Up to 46 | 1.11 (0.98-1.26) | NA | NA |
| Larynx cancer incidence | Low | 1 | 5,657,455 | 509 | Up to 45 | 1.52 (0.73-3.18) | NA | NA |
| Lung cancer incidence | Low | 1 | NR | 3,206 | Up to 46 | 1.13 (1.02-1.25) | NA | NA |
| Melanoma incidence | Low | 2 | NR | 3,630 | Up to 46 | 0.72 (0.65-0.79) | 0.0 (0.765) | NA |
| Breast cancer incidence | Low | 1 | NR | 10,959 | Up to 46 | 0.96 (0.93-1.00) | NA | NA |
| Uterine cervix cancer incidence | Low | 1 | NR | 1,450 | Up to 46 | 1.19 (1.08-1.31) | NA | NA |
| Uterine corpus cancer incidence | Low | 1 | NR | 1,858 | Up to 46 | 0.76 (0.70-0.82) | NA | NA |
| Ovary cancer incidence | Low | 1 | NR | 1,765 | Up to 46 | 0.98 (0.90-1.06) | NA | NA |
| Prostate cancer incidence | Low | 1 | NR | 5,910 | Up to 46 | 0.97 (0.91-1.03) | NA | NA |
| Testis cancer incidence |  | 3 | >222,769.84 | >1,030 | Up to 46 | 0.73 (0.64-0.83) | 0.0 (0.581) | NA |
|  | Low | 2 | NR | 1030 | Up to 46 | 0.73 (0.62-0.86) | 7.1 (0.300) | 0.942 |
|  | High | 1 | 222769.84 | NR | Up to 6 | 0.75 (0.38-1.48) | NA |  |
| Kidney cancer incidence | Low | 1 | NR | 1,380 | Up to 46 | 1.00 (0.86-1.16) | NA | NA |
| Urinary bladder cancer incidence | Low | 1 | NR | 1,990 | Up to 46 | 1.01 (0.89-1.15) | NA | NA |
| Thyroid cancer incidence | Low | 1 | NR | 787 | Up to 46 | 1.04 (0.88-1.22) | NA | NA |
| CNS cancer incidence | Low | 1 | NR | 3,466 | Up to 46 | 1.06 (0.99-1.13) | NA | NA |
| Multiple myeloma | Low | 1 | NR | 680 | Up to 45 | 1.34 (1.08-1.66) | NA | NA |
| Leukemia | Low | 1 | NR | 2,884 | Up to 45 | 1.13 (1.00-1.27) | NA | NA |
| Lymphoma incidence | Low | 2 | NR | 3094 | Up to 45 | 1.05 (0.92-1.20) | 0.0 (0.332) | NA |
| Eye | Low | 1 | 5,657,455 | 774 | Up to 45 | 0.91 (0.51-1.64) | NA | NA |
| Bone cancer incidence | Low | 1 | NR | 259 | Up to 46 | 0.85 (0.63-1.13) | NA | NA |
| Connective and soft tissue cancer incidence | Low | 1 | NR | 565 | Up to 46 | 0.90 (0.73-1.12) | NA | NA |
| **Longer breastfeeding duration compared to shorter duration** | | | | | | | | |
| Overall cancer incidence | Low | 1 | 3,844 | NR | Mean 48.3 | 1.07 (0.89-1.28) | NA | NA |
| Gastric cancer incidence | Low | 1 | 3,844 | NR | Mean 48.3 | 1.22 (0.47-3.15) | NA | NA |
| Colorectal cancer incidence | Low | 1 | 3,844 | NR | Mean 48.3 | 0.86 (0.45-1.63) | NA | NA |
| Breast cancer incidence | Low | 1 | NR | NR | Mean 48.3 | 1.62 (0.89-2.94) | NA | NA |
| Prostate cancer incidence | Low | 1 | NR | NR | Mean 48.3 | 1.43 (0.58-3.52) | NA | NA |
| Overall cancer mortality | Low | 1 | 3,844 | NR | Mean 48.3 | 1.09 (0.86-1.37) | NA | NA |
| Gastric cancer mortality | Low | 1 | 3,844 | NR | Mean 48.3 | 1.43 (0.51-4.01) | NA | NA |
| Colorectal cancer mortality | Low | 1 | 3,844 | NR | Mean 48.3 | 0.96 (0.41-2.21) | NA | NA |
| Breast cancer mortality | Low | 1 | NR | NR | Mean 48.3 | 1.53 (0.61-3.83) | NA | NA |
| Prostate cancer mortality | Low | 1 | NR | NR | Mean 48.3 | 1.34 (0.42-4.22) | NA | NA |

CI, confidence interval; NA, not applicable; NR, not reported; RR, relative risk
